# Supplementary figures and images for: A unified framework to model synaptic dynamics during the sleep–wake cycle
Source: PLoS Biol. 2025 Jun 12;23(6):e3003198. doi: 10.1371/journal.pbio.3003198 (PMC12161594; doi:10.1371/journal.pbio.3003198)

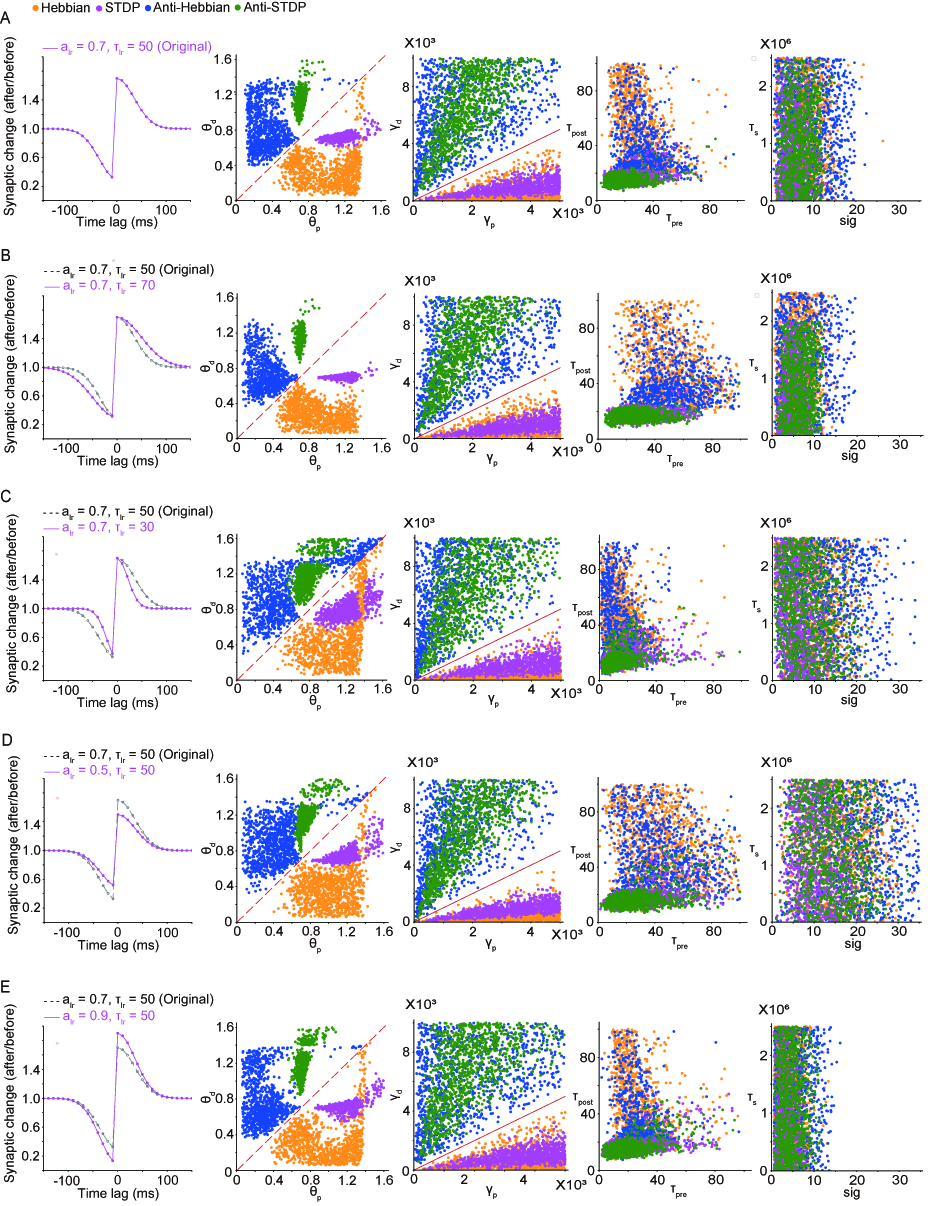

Supplement: S1 Fig — Distributions for parameter sets for four types of synaptic learning rules obtained by different fitting curves. A total of 1,000 parameter sets whose sum of squared errors (SSE) between the analytical result and fitted Gaussian curves was less than thresholds were selected. The threshold was 0.45 in alr = 0.9, τlr = 50 (E) or 0.25 in other conditions (A–D). (A) The original one. The plots in axes of thresholds and amplitudes were the same as in Fig 1F. (B–E) Parameter sets were collected by fitting to gaussian curves with different amplitudes (alr) and time constants (τlr) from original one. The ranges of parameters are shown in S2 Table. (TIF) [file pbio.3003198.s001.tif]

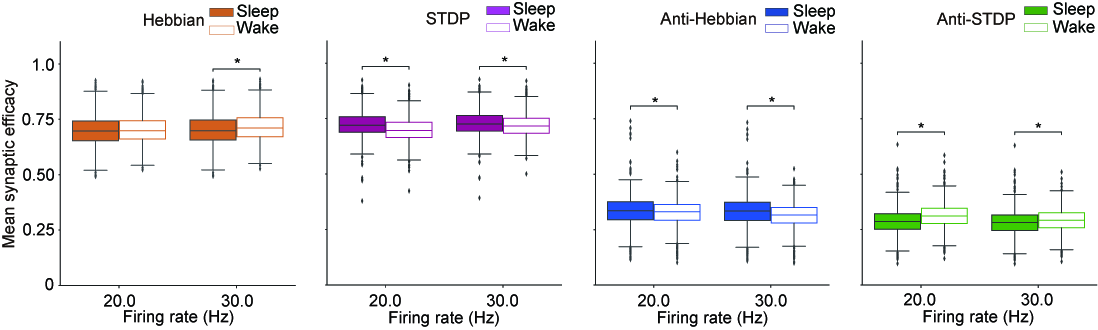

Supplement: S2 Fig — Box plots for mean synaptic efficacy under four different types of synaptic learning rules at higher mean firing rates (n = 1,000 for each firing rate, n represents the number of synaptic learning rules). The parameter sets for synaptic learning rules were the same as in Fig 1H. The sleep-like firing patterns were generated by sampling from the lognormal distributions for Up-state duration and Down-state duration (log10 (mean Up-state duration) = 2.7 and log10 (mean Down-state duration) = 2.7, SD was calculated according to the linear regression analysis based on in vivo data (S18 Fig)). Initial synaptic efficacies in all the synapses were 0.5 and synaptic efficacies were simulated for 6 min. Synaptic efficacies for the last 2 min were averaged and compared between sleep-like and wake-like firing patterns. The whiskers above and below of box plots show minimal to maximal values. The box extends from the 25th to the 75th percentile and the middle line indicates the median. Bayesian statistical analysis was performed using Markov Chain Monte Carlo method to infer posterior distributions of average differences in mean synaptic efficacy between sleep-like firing patterns and wake-like firing patterns. Asterisks (*) indicate 95% CIs do not include zero. The data underlying the graphs shown in the figure can be found in Table A in S2 Data. The 95% CIs for the distributions of average differences are shown in Table F in S3 Data. (TIF) [file pbio.3003198.s002.tif]

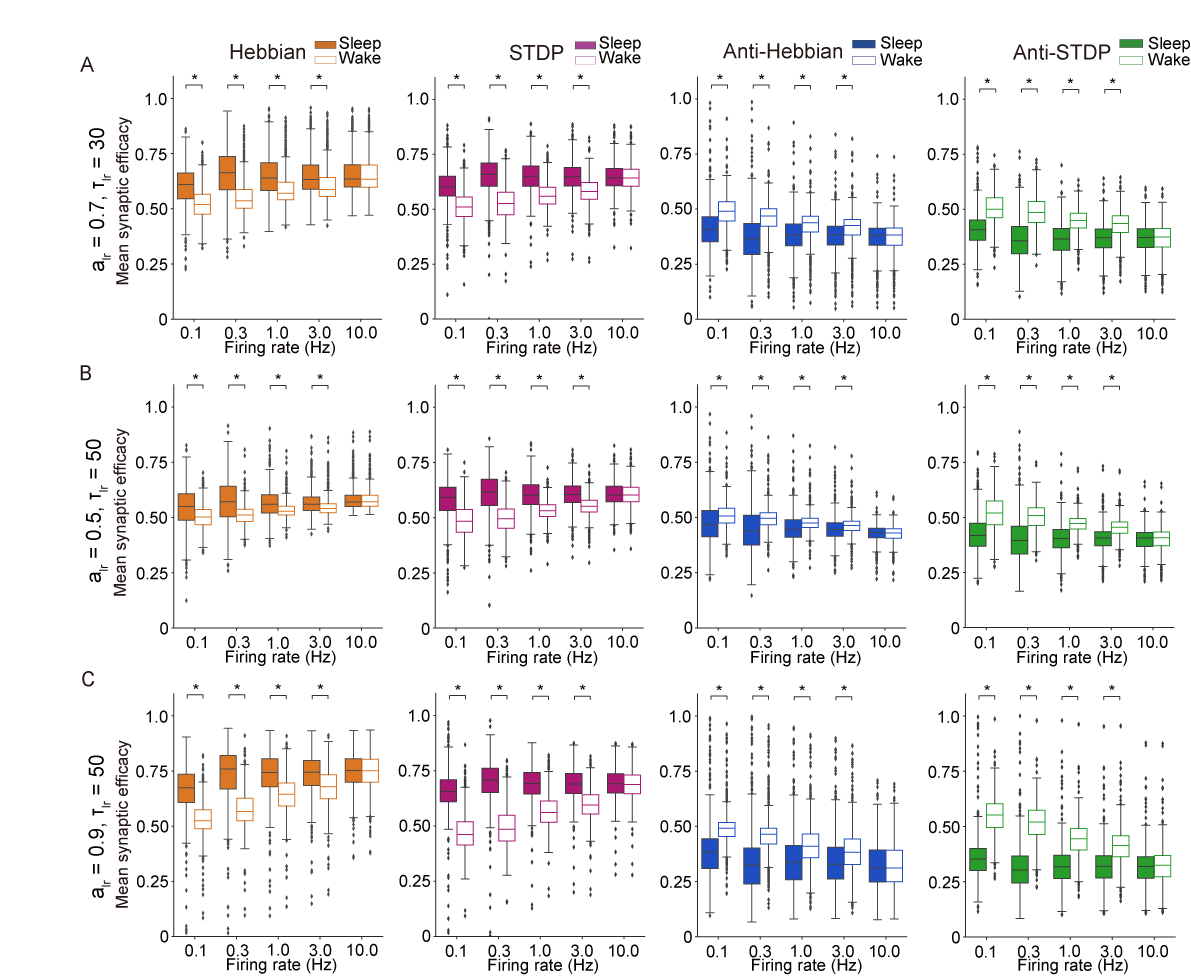

Supplement: S3 Fig — (A–C) Box plots for mean synaptic efficacy under synaptic learning rules in different parameters for fitting curves (n = 1,000 for each firing rate, n represents the number of synaptic learning rules). The results of alr=0.7, τlr=30 (A), alr=0.5, τlr=50 (B), and alr=0.9, τlr=50 (C) are shown. The parameter sets for synaptic learning rules were the same as in S1 Fig. The sleep-like firing patterns were the same as in Fig 1H. Initial synaptic efficacies in all the synapses were 0.5 and synaptic efficacies were simulated for 6 min. Synaptic efficacies for the last 2 min were averaged and compared between sleep-like and wake-like firing patterns. The whiskers above and below of box plots show minimal to maximal values. The box extends from the 25th to the 75th percentile and the middle line indicates the median. Bayesian statistical analysis was performed using Markov Chain Monte Carlo method to infer posterior distributions of average differences in mean synaptic efficacy between sleep-like firing patterns and wake-like firing patterns. Asterisks (*) indicate 95% CIs do not include zero. The data underlying the graphs shown in the figure can be found in Tables B–D in S2 Data. The 95% CIs for the distributions of average differences are shown in Tables G-I S3 Data. (TIF) [file pbio.3003198.s003.tif]

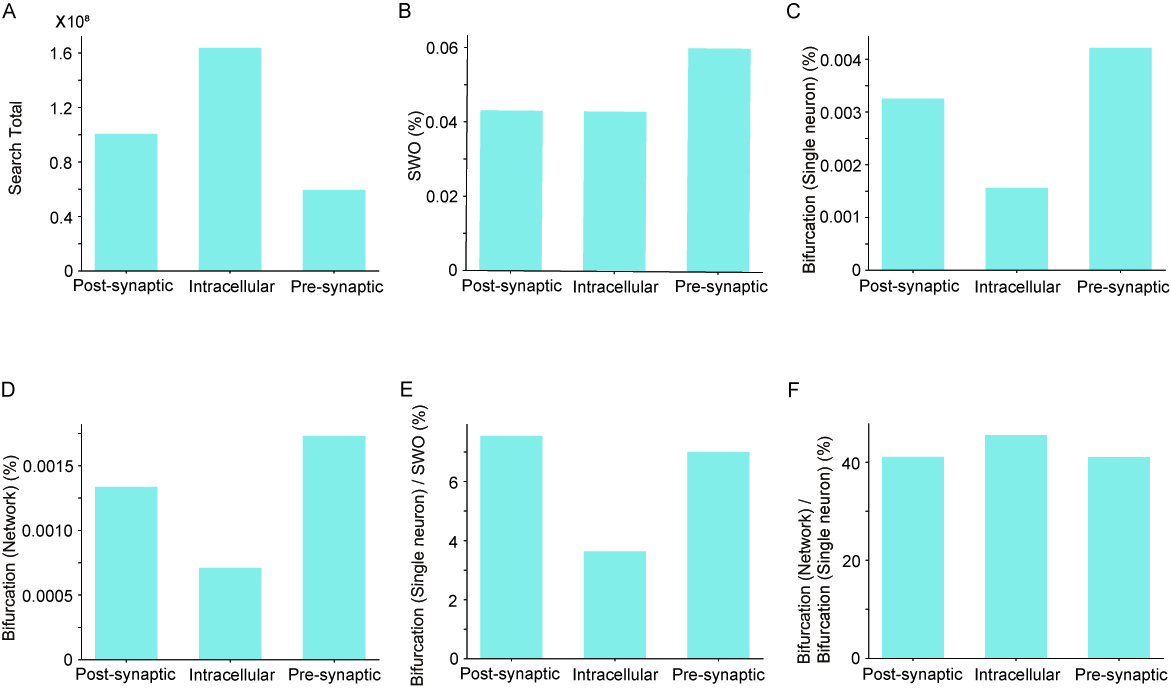

Supplement: S4 Fig — (A–F) The results of parameter search for SWO and bifurcation analysis in single and multiple neurons of the Hodgkin–Huxley-based model is shown by three types of bifurcation models. The longitudinal axes represent the number of total parameter sets searched (A), the percentage of parameter sets generating SWO (B), the percentage of parameter sets bifurcating from wake-like to sleep-like firing patterns in a single neuron (bifurcation (single neuron)) (C), the percentage of parameter sets bifurcating from wake-like to sleep-like firing patterns as a network (bifurcation (network)) (D), ratio of bifurcation (single neuron) to SWO (E), ratio of bifurcation (network) to bifurcation (single neuron) (F). The data underlying the graphs shown in the figure can be found in Table E in S2 Data. (TIF) [file pbio.3003198.s004.tif]

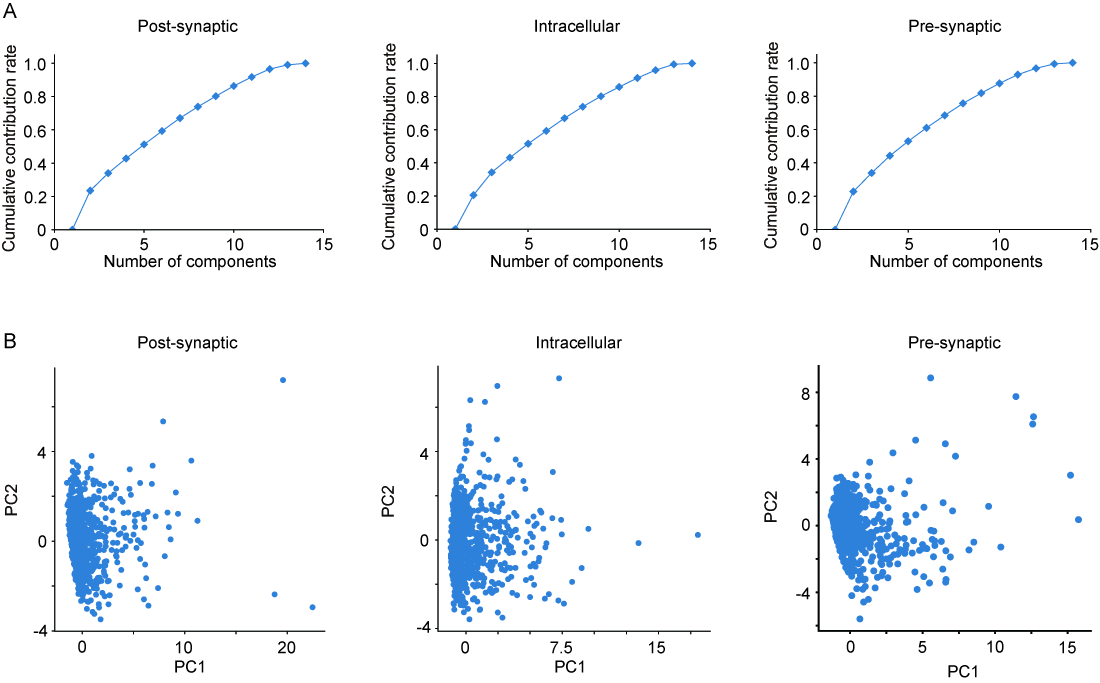

Supplement: S5 Fig — Principal component analysis (PCA) was conducted for the parameter sets that bifurcated form sleep-like to wake-like firing patterns as a network obtained in bifurcation analysis in each Hodgkin–Huxley-based network model. (A) Cumulative contribution ratio of eigen values when PCA applied to the parameter sets. (B) Projection of the parameter sets onto their first two principal components in each model (n = 1,344, 1,202, and 1,092 in the post-synaptic, intracellular and pre-synaptic bifurcation models, respectively). (TIF) [file pbio.3003198.s005.tif]

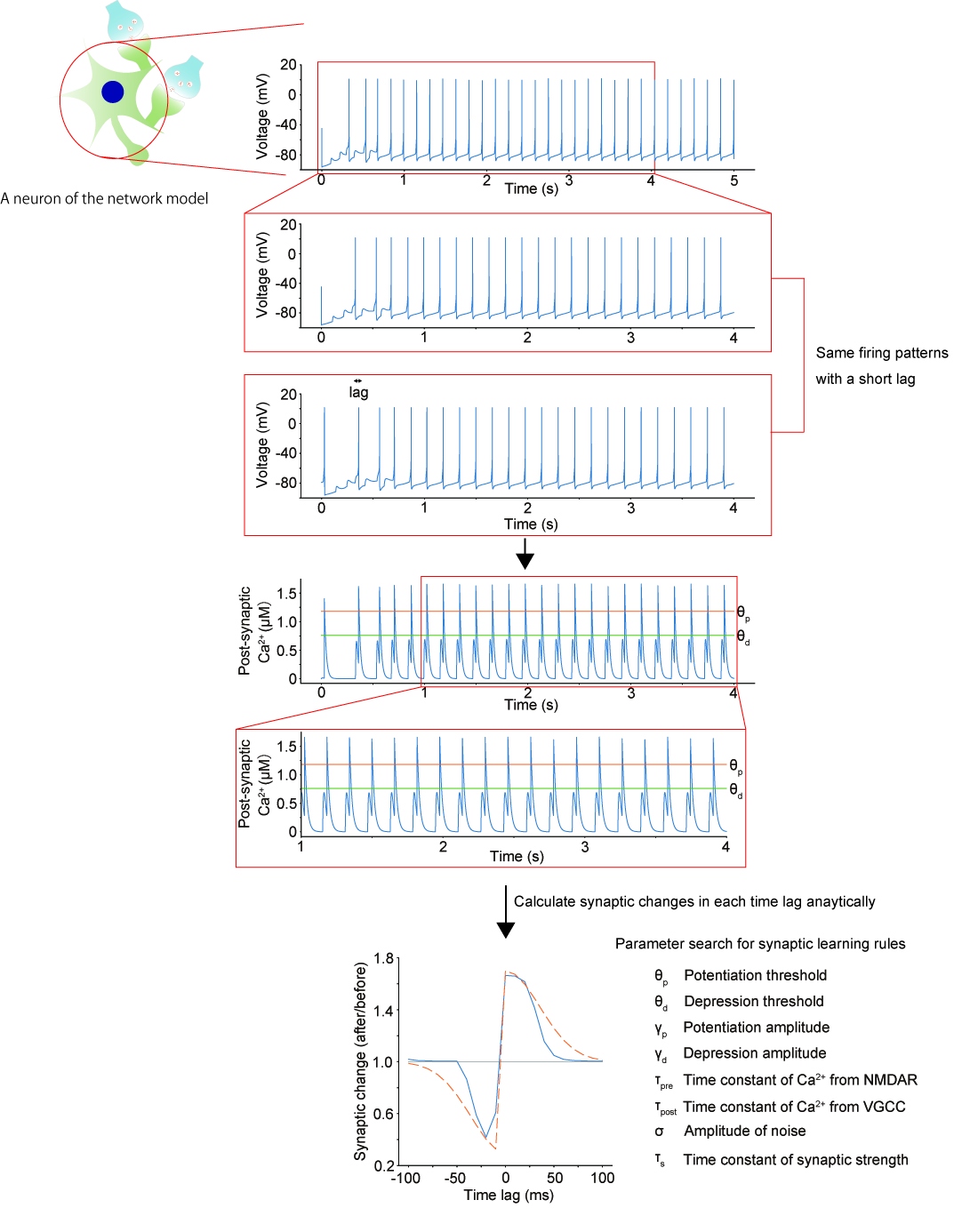

Supplement: S6 Fig — The details are shown in “Materials and methods, Parameter search for synaptic learning rules in Hodgkin–Huxley-based network models”. A 4-s time series data for membrane potentials of a single neuron in a network model during wake-like states were selected and duplicated with a short delay in each time lag. Parameter sets for synaptic learning rules were randomly generated and the periods that post-synaptic Ca2+ exceeded θp or θdin 3 s (from one to four seconds) were calculated respectively. Then, the periods spent above each threshold were multiplied by 20 to obtain the total values for 60 s for the purpose of speeding up the calculations. Synaptic changes in each time lag were analytically calculated and the parameter sets of which the SSE between analytical results and the Gaussian fitting curves was less than 0.6 were selected. (TIF) [file pbio.3003198.s006.tif]

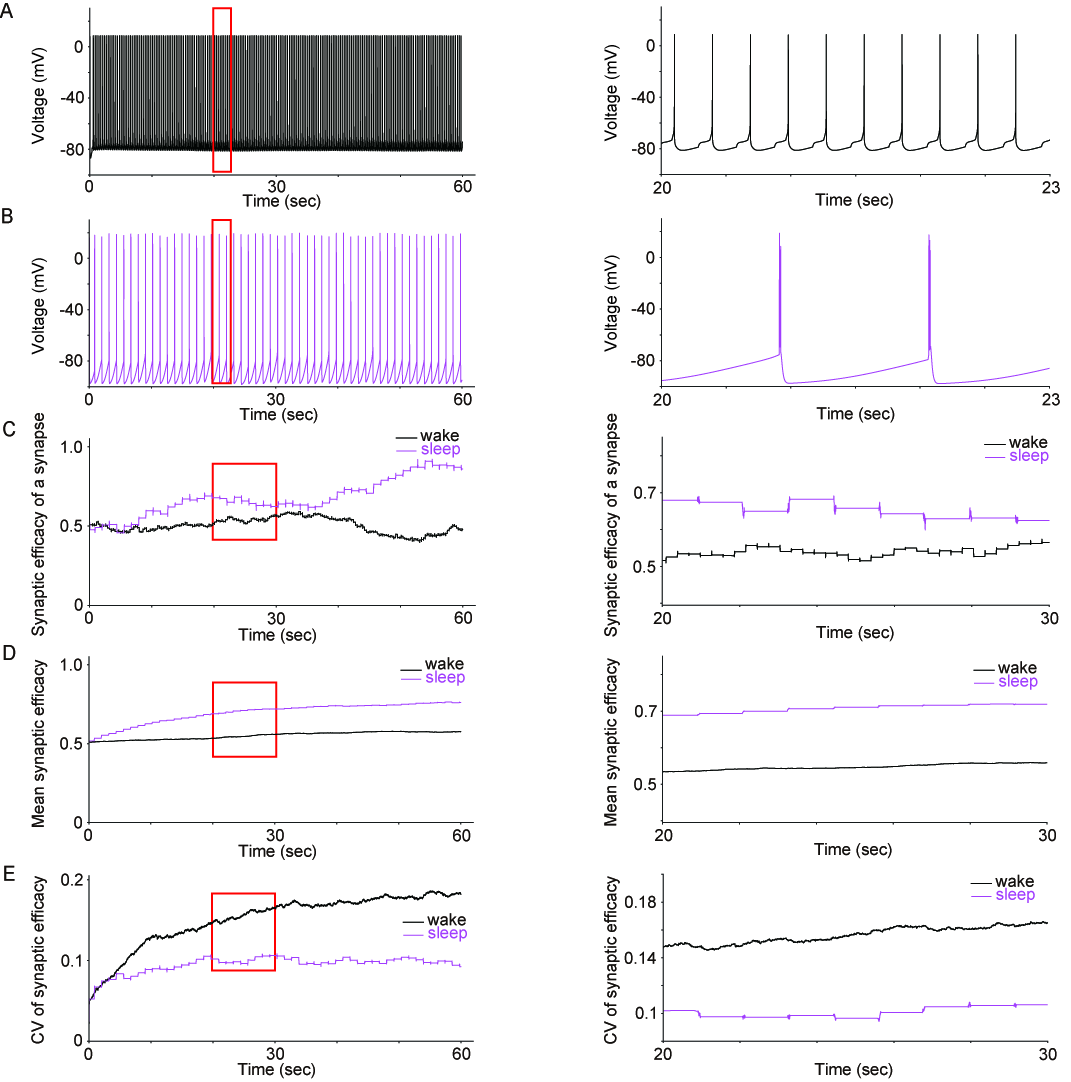

Supplement: S7 Fig — (A–E) Time changes in membrane potential and synaptic efficacy were calculated in a representative intracellular bifurcation model. The structure and parameter set for the channel and receptor conductance of network model was the same as in Fig 3C and the parameter set for STDP learning rule is shown in S8 Table. Initial synaptic efficacies of all synapses were 0.5. Simulations were conducted for 60 s. Time changes in membrane potential during wake-like patterns (A), membrane potential in sleep-like patterns (B), synaptic efficacy of a representative synapse (C), mean synaptic efficacy (D), and CV of synaptic efficacy (E) are shown. Right figures show enlarged graphs for red rectangles of left figures. (TIF) [file pbio.3003198.s007.tif]

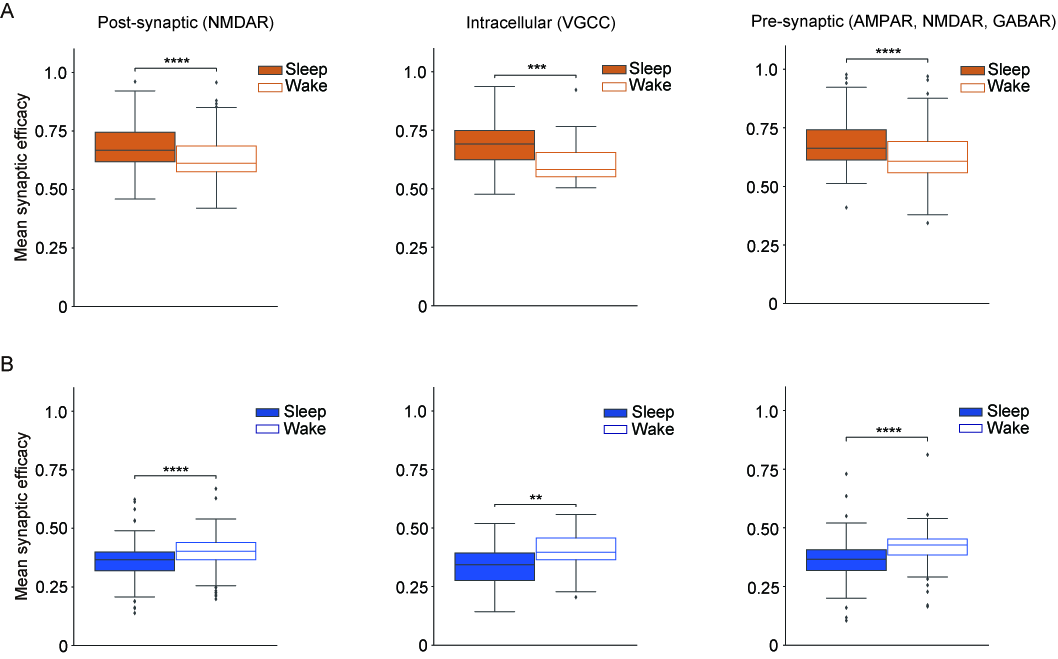

Supplement: S8 Fig — (A, B) Box plots for mean synaptic efficacy in sleep-like and wake-like firing patterns under Hebbian (A) and Anti-Hebbian (B) by three types of network models (n = 192, 42 and 151 for Hebbian and n = 143, 38 and 123 for Anti-Hebbian in post-synaptic, intracellular, and pre-synaptic bifurcation models respectively, n represents the number of parameter sets for the network models). The structure and parameter set for channel or receptor conductance of network models were the same as in Fig 3E and 3F. A parameter set for the synaptic learning rule was assigned to each network model. Synaptic efficacy was compared assuming the almost close firing rates between sleep-like and wake-like states. Initial synaptic efficacies of all synapses were 0.5. Simulations were conducted for 60 s and synaptic efficacy and CV were averaged over the period from 10 to 60 s. The whiskers above and below of box plots show minimal to maximal values. The box extends from the 25th to the 75th percentile and the middle line indicates the median. *p < 0.05, **p < 0.01, ***p < 0.001, ****p < 0.0001, Student t test was applied. The data underlying the graphs shown in the figure can be found in Table F in S2 Data. (TIF) [file pbio.3003198.s008.tif]

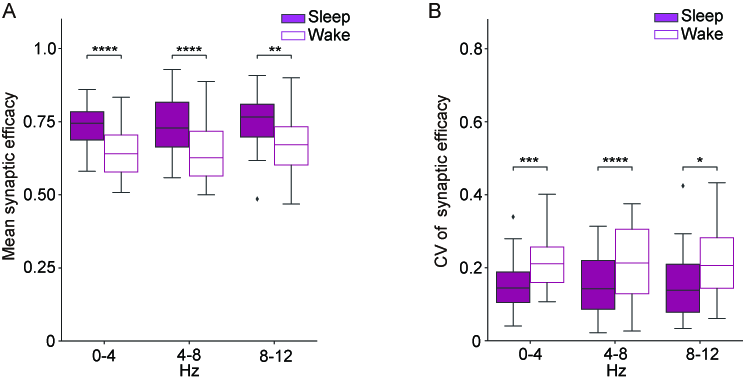

Supplement: S9 Fig — (A, B) Mean (A) and CV (B) of synaptic efficacy in the post-synaptic bifurcation models under STDP were compared between sleep-like and wake-like firing patterns by different mean firing rates. The results of simulations for models bifurcated by the post-synaptic mechanism in Fig 3E were classified into three groups (0–4 Hz, 4–8 Hz and 8–12 Hz) by mean firing rates during wake-like firing patterns (n = 35, 106 and 29 for each firing rate group, n represents the number of parameter sets for the network models). The box extends from the 25th to the 75th percentile and the middle line indicates the median. *p < 0.05, **p < 0.01, ***p < 0.001, ****p < 0.0001, Student t test was applied. The data underlying the graphs shown in the figure can be found in Tables G and H in S2 Data. (TIF) [file pbio.3003198.s009.tif]

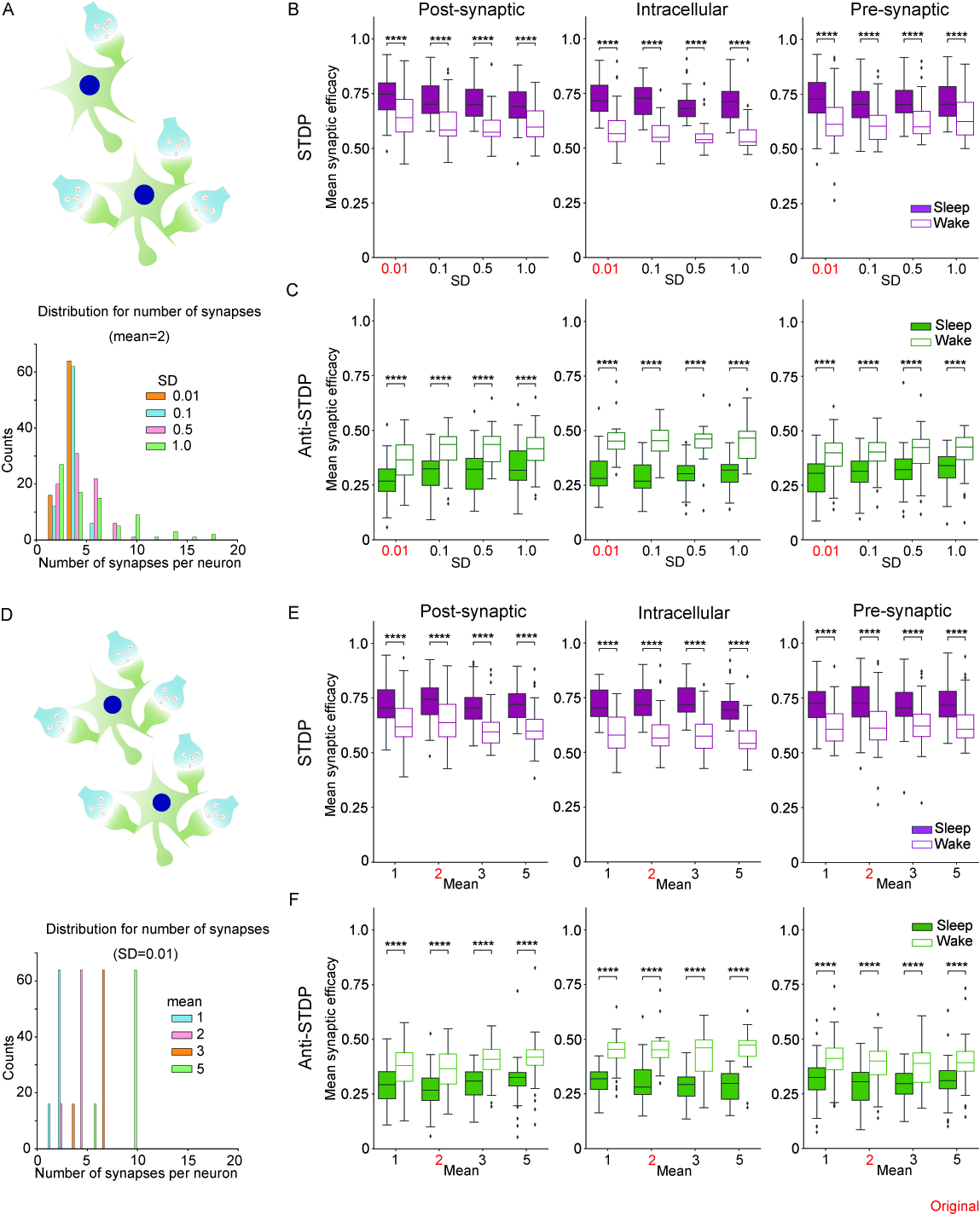

Supplement: S10 Fig — (A) Schematic illustration and histogram for lognormal distributions of the number of synapses per neuron in different SDs. (B, C) Box plots for mean synaptic efficacy during sleep-like and wake-like firing patterns under STDP (n = 191, 74, 46, 43, n = 52, 34, 26, 25, and n = 150, 83, 56, 48 for each value of SD in post-synaptic, intracellular and pre-synaptic bifurcation models, respectively) (B) and Anti-STDP (n = 121, 82, 57, 49, n = 36, 34, 34, 31, and n = 119, 90, 61, 58 for each value of SD in post-synaptic, intracellular and pre-synaptic bifurcation models, respectively) (C) in different SDs for lognormal distributions. (D) Schematic illustration and histogram for lognormal distributions of the number of synapses per neuron in different means. (E, F) Box plots for mean synaptic efficacy during sleep-like and wake-like firing patterns under STDP (n = 89, 191, 85, 83, n = 36, 52, 34, 34, and n = 99, 150, 90, 88 for each value of mean in post-synaptic, intracellular and pre-synaptic bifurcation models, respectively) (E) and Anti-STDP (n = 83, 121, 85, 77, n = 36, 36, 35, 38, and n = 101, 119, 93, 91 for each value of mean in post-synaptic, intracellular and pre-synaptic bifurcation models, respectively.) (F) in different means for lognormal distributions. (B, C, E, F) A parameter set for the synaptic learning rule was assigned to each network model. Synaptic efficacy was compared assuming the almost close firing rates between sleep-like and wake-like states. Initial synaptic efficacies of all synapses were 0.5. Simulations were conducted for 60 s and synaptic efficacy and CV were averaged over the period from 10 to 60 s. The whiskers above and below of box plots show minimal to maximal values. The box extends from the 25th to the 75th percentile and the middle line indicates the median. *p < 0.05, **p < 0.01, ***p < 0.001, ****p < 0.0001, Student t test was applied. The parameter sets for channel and receptor conductance of network models and synaptic learning r [file pbio.3003198.s010.tif]

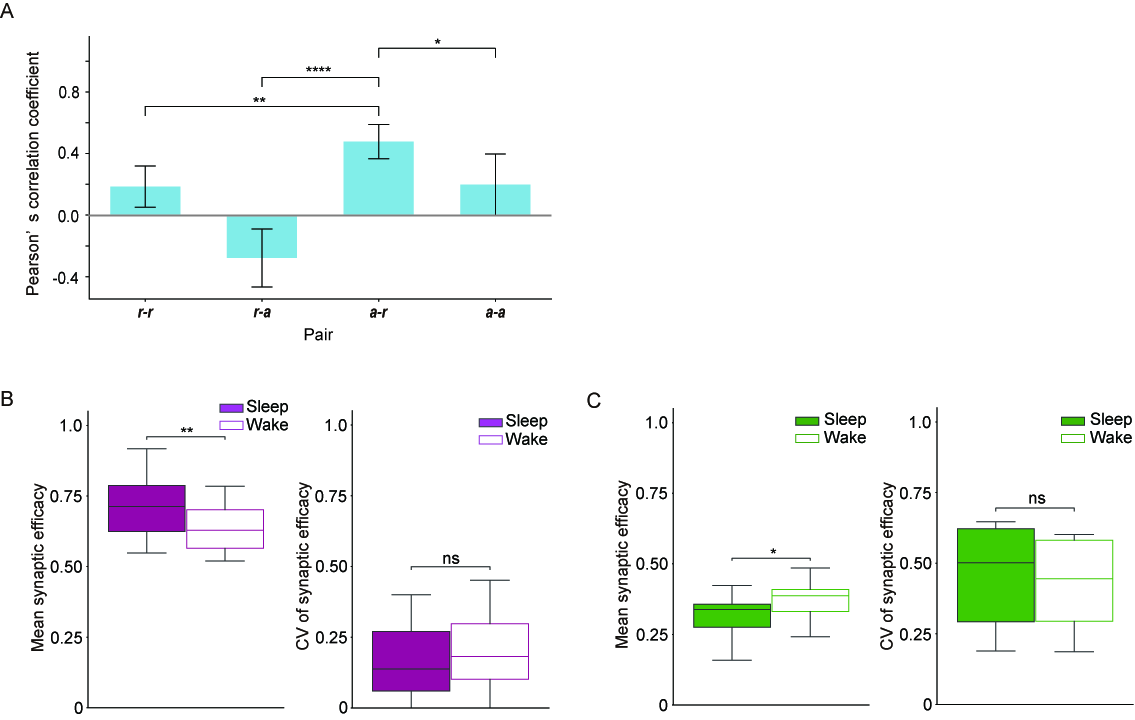

Supplement: S11 Fig — (A) Pearson’s correlation coefficients were calculated in combinations of r and a with multiple parameter sets for Hodgkin–Huxley-based network models. NMDAR conductance was updated by r or a and parameters for sleep–wake dynamics were optimized by Pearson’s correlation coefficients between Process S and r or a (For example, r-a means that conductance was updated by r and parameters for sleep–wake dynamics were optimized by Pearson’s correlation coefficients between Process S and a). The network structures and initial values for variables (a, r and ξ) were the same as in Fig 4. Initial synaptic efficacies of all synapses were 0.5 and the simulations were conducted for 300 s. n = 14, 14, 15, and 15 for r–r, r–a, a–r, and a–a pair, respectively. *p < 0.05, **p < 0.01, ***p < 0.001, ****p < 0.0001, Student t test was applied. The data underlying the graphs shown in the figure can be found in Table K in S2 Data. (B, C) Box plots for mean and CV of synaptic efficacy during sleep-like and wake-like periods in multiple parameter sets for network models with sleep–wake dynamics under STDP (n = 27) (B) and Anti-STDP (n = 12) (C). Initial values for variables (a, r and ξ) were the same as in the representative models in Fig 4. Initial synaptic efficacies of all synapses were 0.5 and the simulations were conducted for 300 s. NMDAR conductance was updated by a and optimized by Pearson’s correlation coefficients between Process S and r. The whiskers above and below of box plots show minimal to maximal values. The box extends from the 25th to the 75th percentile and the middle line indicates the median. *p < 0.05, **p < 0.01, ***p < 0.001, ****p < 0.0001, Student t test was applied. The data underlying the graphs shown in the figure can be found in Tables L and M in S2 Data. (TIF) [file pbio.3003198.s011.tif]

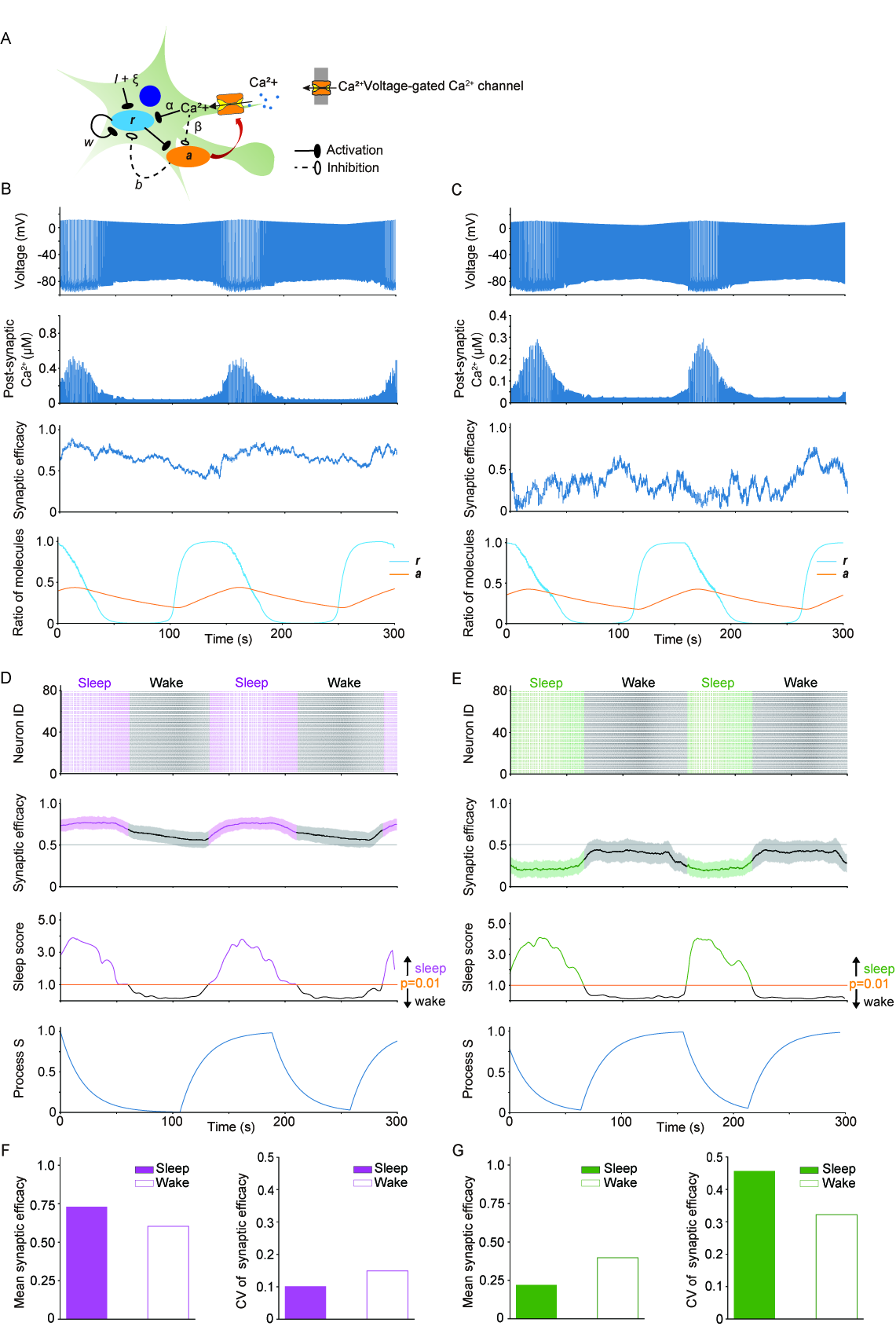

Supplement: S12 Fig — VGCC conductance was updated by a and parameters for the sleep–wake dynamics model were optimized by Pearson’s correlation coefficient between Process S and r. Initial synaptic efficacies of all synapses were 0.5. The simulations were started from a sleep-like state and conducted for 500 s. The network structure was the same as in Fig 4B. The parameter set for channel or receptor conductance of network models, synaptic learning rules and sleep–wake dynamics and initial values for variables in a representative model are shown in S5–S8 Tables. (A) Schematic illustration of the model for sleep–wake dynamics in the intracellular bifurcation mechanism. Ca2+ in a cell body activates the initial state of CaMKII represented by r. (B, C) Time changes in membrane potential of a neuron and post-synaptic Ca2+, synaptic efficacy and ratio of two phosphorylated states of kinases (r and a) of a synapse in representative network models under STDP (B) and Anti-STDP (C). The results from 200 to 500 s are shown. (D, E) Raster plots and time changes in mean synaptic efficacy, sleep score and Process S in representative network models under STDP (D) and Anti-STDP (E). The shadow in time changes in mean synaptic efficacy represents SD. The network was considered to be in the sleep-like or wake-like states if the sleep score was above or below the threshold, respectively (the threshold is the value of sleep score where p = 0.01, see “Materials and methods, Evaluation of synchronization and desynchronization in Hodgkin–Huxley-based network models”). The results of 200–500 s are shown. (F, G) Mean and CV of synaptic efficacy during the periods of sleep-like and wake-like states in representative network models under STDP (F) and Anti-STDP (G). The data underlying the graphs shown in the figure can be found in Table N in S2 Data. (TIF) [file pbio.3003198.s012.tif]

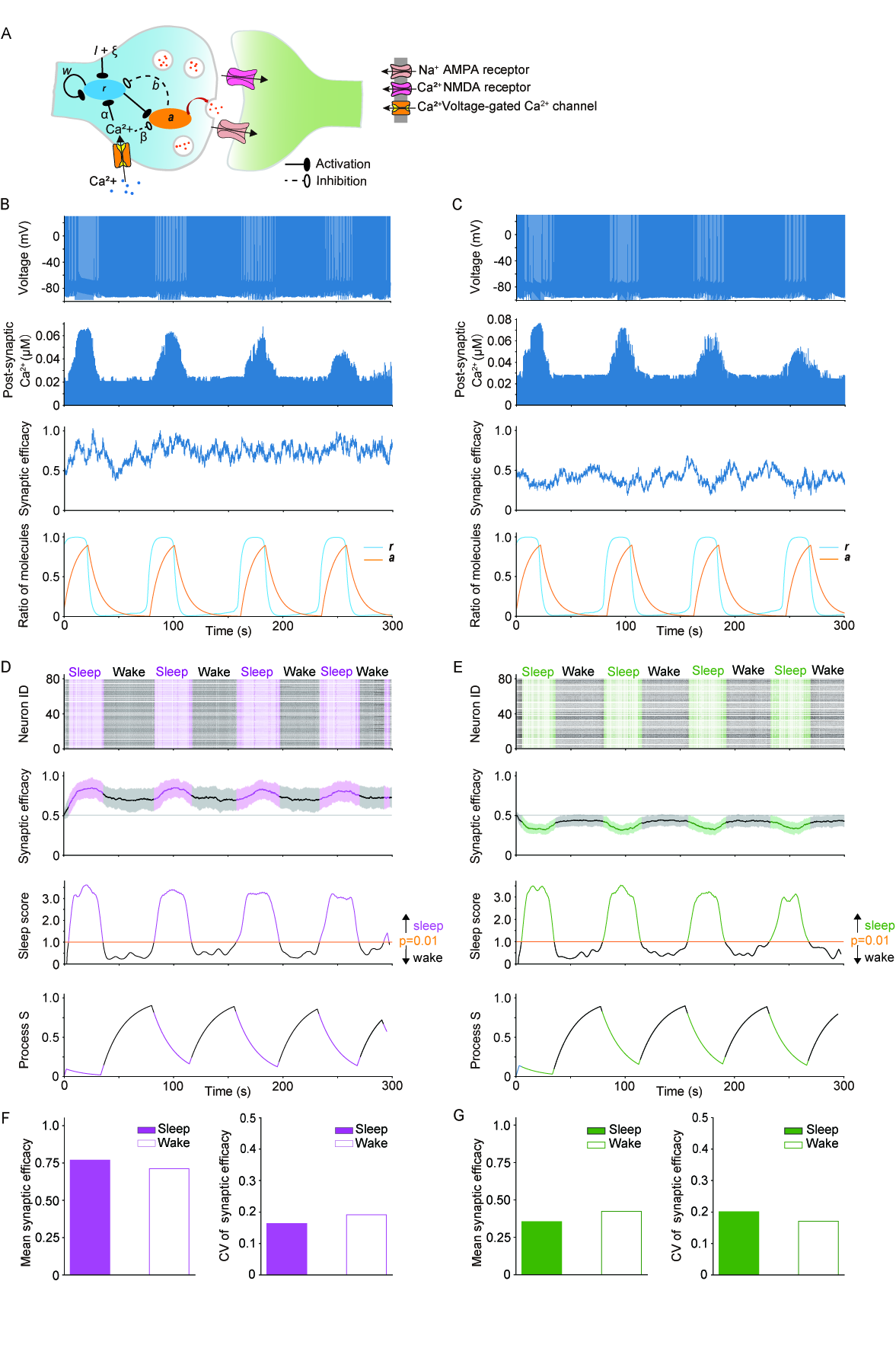

Supplement: S13 Fig — Coefficients for pre-synaptic activations were updated by a and parameters for the sleep–wake dynamics model were optimized by Pearson’s correlation coefficient between Process S and r. Initial synaptic efficacies of all synapses were 0.5. The simulations were started from a wake-like state and conducted for 300 s. The network structure was the same as in Fig 4B. The parameter set for channel or receptor conductance of network models, synaptic learning rules and sleep–wake dynamics and initial values for variables in a representative model are shown in S5–S8 Tables. (A) Schematic illustration of the model for sleep–wake dynamics in the intracellular bifurcation mechanism. Ca2+ in a pre-synaptic neuron activates the initial state of CaMKII represented by r. (B, C) Time changes in membrane potential of a neuron and post-synaptic Ca2+, synaptic efficacy and ratio of two phosphorylated states of kinases (r and a) of a synapse in representative network models under STDP (B) and Anti-STDP (C). (D, E) Raster plots and time changes in mean synaptic efficacy, sleep score and Process S in representative network models under STDP (D) and Anti-STDP (E). The shadow in time changes in mean synaptic efficacy represents SD. The network was considered to be in the sleep-like or wake-like states if the sleep score was above or below the threshold, respectively (the threshold is the value of sleep score where p = 0.01, see “Materials and methods, Evaluation of synchronization and desynchronization in Hodgkin–Huxley-based network models”). (F, G) Mean and CV of synaptic efficacy during the periods of sleep-like and wake-like states in representative network models under STDP (F) and Anti-STDP (G). The data underlying the graphs shown in the figure can be found in Table O in S2 Data. (TIF) [file pbio.3003198.s013.tif]

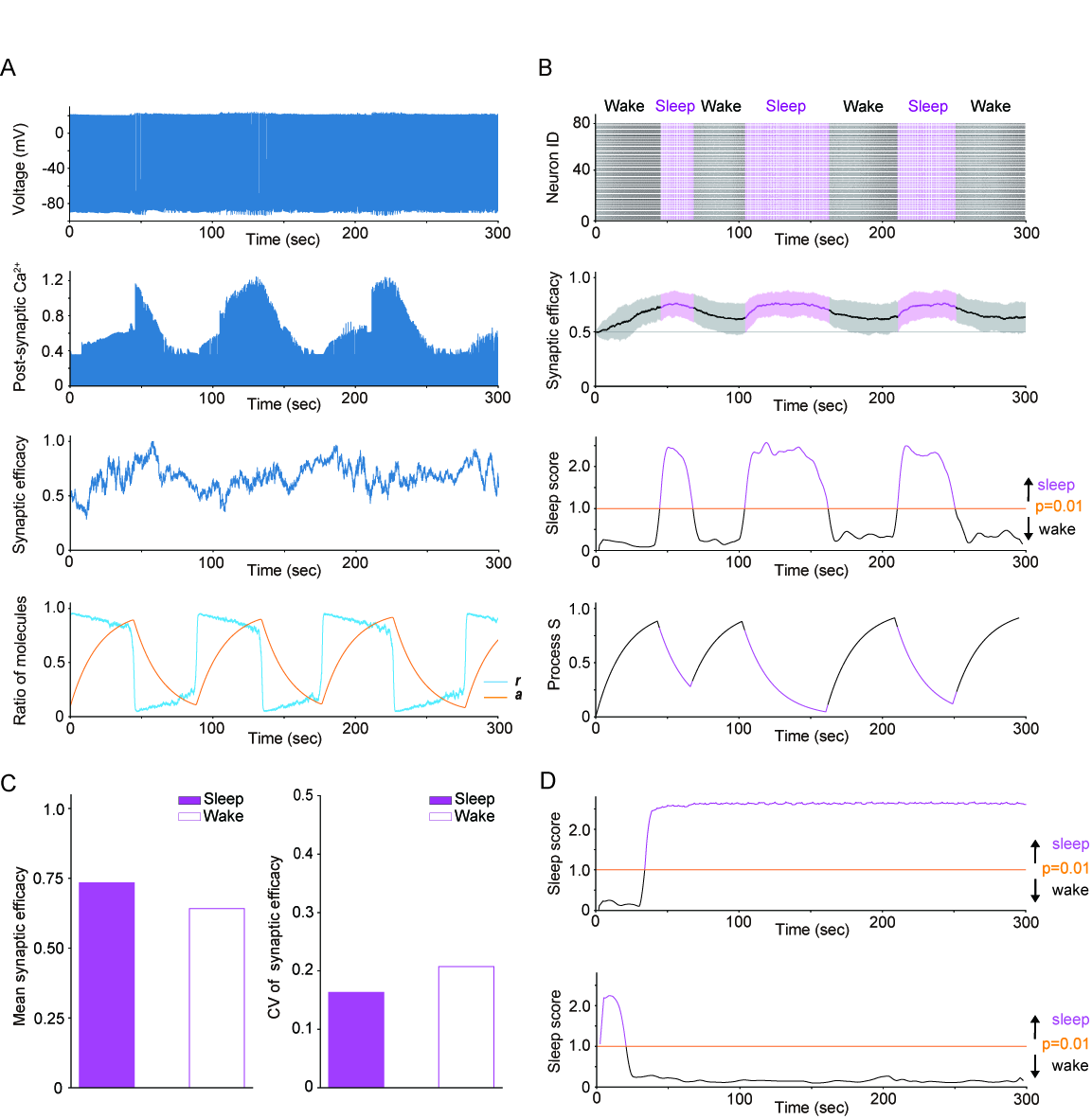

Supplement: S14 Fig — The model for sleep–wake dynamics can represent multiple regimes. In the oscillatory regime (Fig 4), activity alternates between sleep-like and wake-like states. In the bistable regime, although sleep-like and wake-like states are relatively stable, sufficiently large noises induces alternations between two states, resulting in variable durations of two states. Synaptic efficacy was calculated in a representative network model bifurcated by the post-synaptic mechanism with sleep–wake dynamics and STDP learning rule in the bistable regime. The conductance of NMDAR was updated by a and the simulations were optimized by Pearson’s correlation coefficients between Process S and r. Initial synaptic efficacies of all synapses were 0.5. The simulations were started from a wake-like state and conducted for 300 s. The network structure was the same as in Fig 4B. The parameter set for channel or receptor conductance of network models, synaptic learning rules and sleep–wake dynamics and initial values for variables in a representative model are shown in S5–S8 Tables. (A) Time changes in membrane potentials, post-synaptic Ca2+, synaptic efficacy, and ratio of two phosphorylated states of kinases (r and a) in a single neuron of a representative network model. (B) Raster plots, time changes in mean synaptic efficacy, sleep score, and Process S in a representative network model. The shadow in time changes in mean synaptic efficacy represents SD. The network was considered to be in the sleep-like or wake-like states if the sleep score was above or below the threshold, respectively (the threshold is the value of sleep score where p = 0.01, see “Materials and methods, Evaluation of synchronization and desynchronization in Hodgkin–Huxley-based network models”). (C) Mean and CV of synaptic efficacy during sleep-like and wake-like periods in a representative network model. The data underlying the graphs shown in the figure can be found in Table P in S2 Data. (D) The results of simulation [file pbio.3003198.s014.tif]

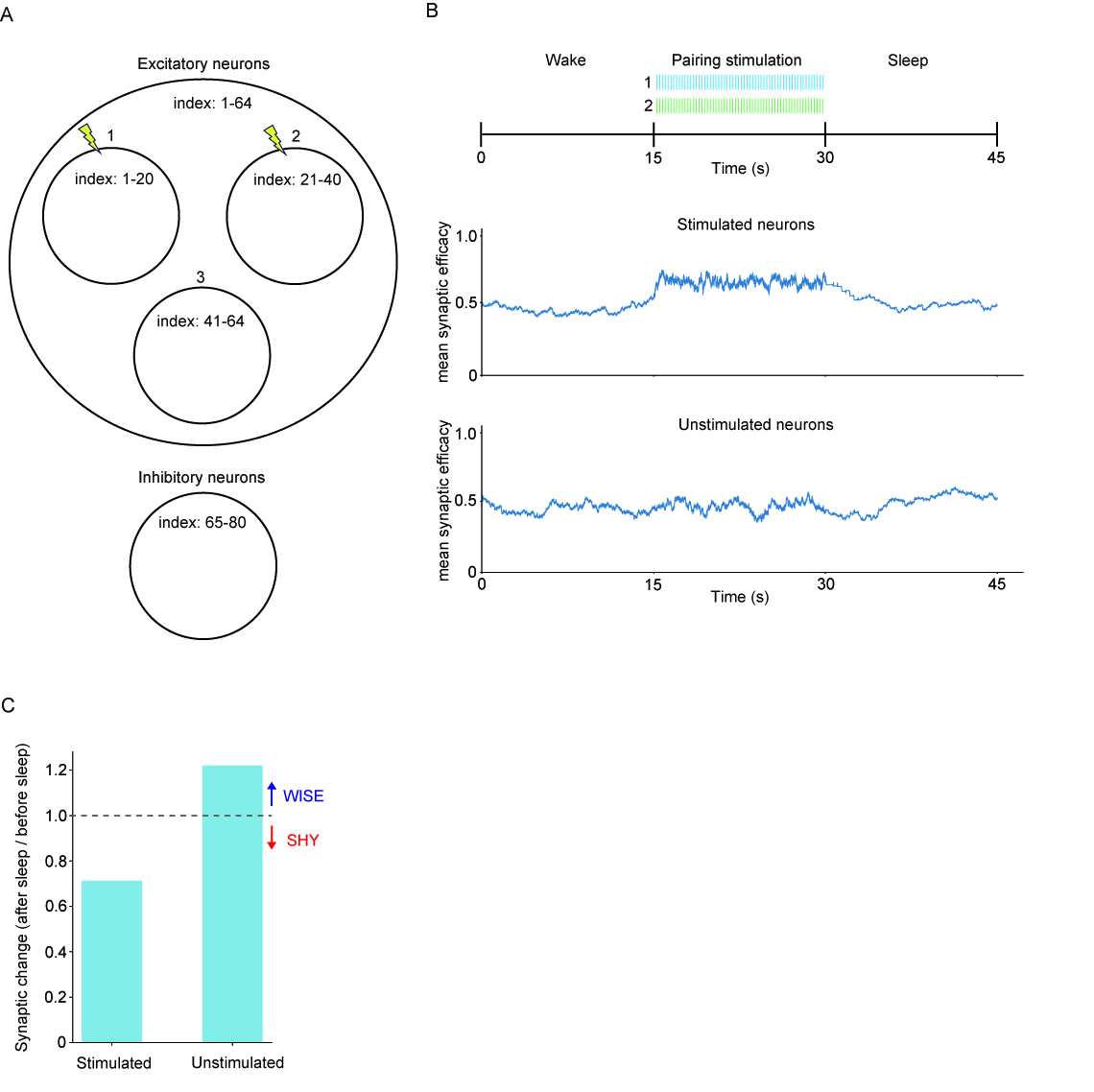

Supplement: S15 Fig — Synaptic efficacy was calculated in a representative network model bifurcated by the intracellular mechanism under STDP. Parameter sets for the channel or receptor conductance and the synaptic learning rule are shown in S5 and S8 Tables, respectively. The VGCC conductance was multiplied by 10−0.4 and 10−0.1 to its original value to generate wake-like and sleep-like firing patterns. Stimulations were applied for 15 s at 20 Hz after wake-like firing patterns. The stimulation was optimized by changing its waveforms and rates so that the potentiation of synaptic efficacy between stimulated groups were observed (see “Materials and methods, Calculation of synaptic efficacy under synaptic learning rules in Hodgkin–Huxley-based network models including stimulation during the wakefulness”). Initial synaptic efficacies of all synapses were 0.5. The network structure was the same as in Fig 4B. (A) Schematic illustration for grouping excitatory neurons. Group 1 and 2 were stimulated. (B) Time changes in mean synaptic efficacy of stimulated neurons and unstimulated neurons. (C) Ratio of mean synaptic efficacy after and before sleep-like firing patterns in stimulated and unstimulated neurons. The data underlying the graphs shown in the figure can be found in Table Q in S2 Data. (TIF) [file pbio.3003198.s015.tif]

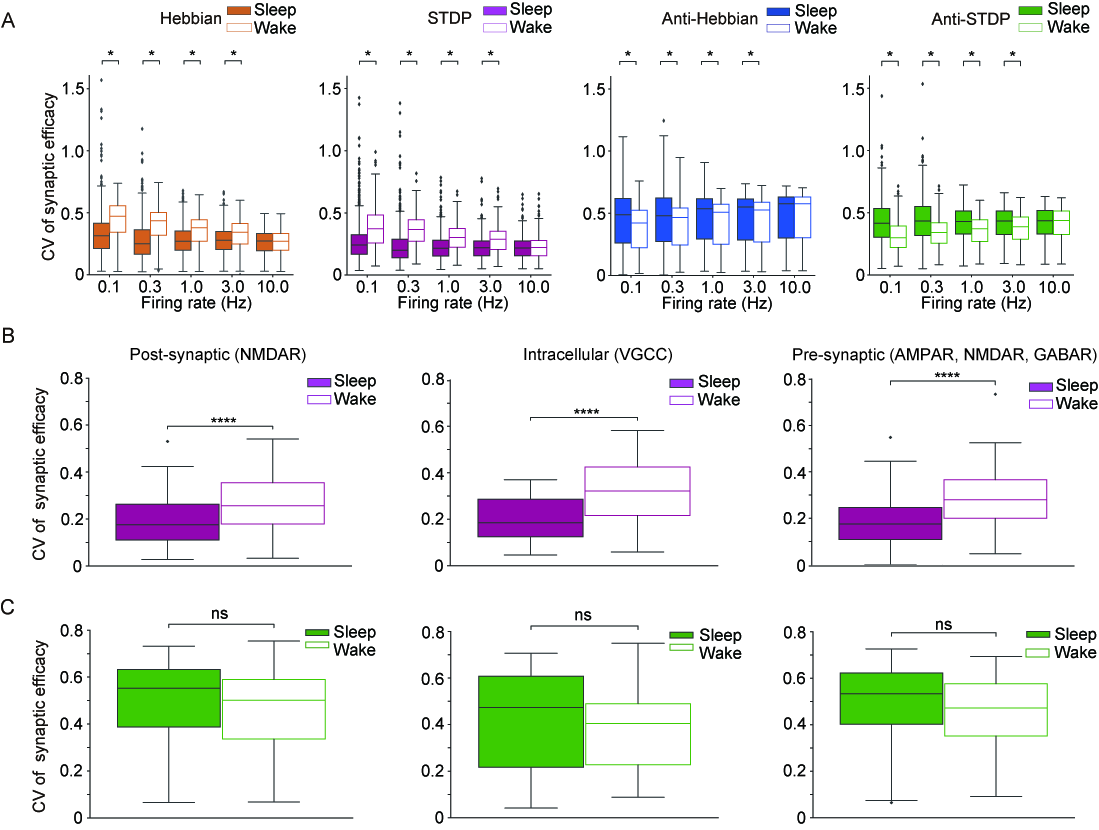

Supplement: S16 Fig — (A) Box plots for CV of synaptic efficacy in sleep-like and wake-like firing patterns by synaptic learning rules and mean firing rates in simple model (n = 1,000 for each firing rate, n represents the number of synaptic learning rules). The network structure, firing patterns and parameters for synaptic learning rules are the same as in Fig 1H. Initial synaptic efficacies in all the synapses were 0.5 and synaptic efficacies were simulated for 6 min. Synaptic efficacies for the last 2 min were averaged and compared between sleep-like and wake-like firing patterns. The whiskers above and below of box plots show minimal to maximal values. The box extends from the 25th to the 75th percentile and the middle line indicates the median. Bayesian statistical analysis was performed using Markov Chain Monte Carlo method to infer posterior distributions of average differences in mean synaptic efficacy between sleep-like firing patterns and wake-like firing patterns. Asterisks (*) indicate 95% CIs do not include zero. The data underlying the graphs shown in the figure can be found in Table R in S2 Data. The 95% CIs for the distributions of average differences are shown in Table J in S3 Data. (B, C) Box plots for CV of synaptic efficacy during sleep-like and wake-like firing patterns under STDP (B) and Anti-STDP (C) in Hodgkin–Huxley-based network models (n = 191, 52 and 150 for STDP and n = 121, 36 and 119 for Anti-STDP in post-synaptic, intracellular, and pre-synaptic bifurcation models respectively. n represents the number of parameter sets for the network models). The network structure, firing patterns and parameters for synaptic learning rules are the same as in Fig 3E and 3F. Synaptic efficacy was compared assuming the almost close firing rates between sleep-like and wake-like states. Initial synaptic efficacies of all synapses were 0.5. Simulations were conducted for 60 s, and synaptic efficacy and CV were averaged over the period from 10 to 60 s. The whiskers above and bel [file pbio.3003198.s016.tif]

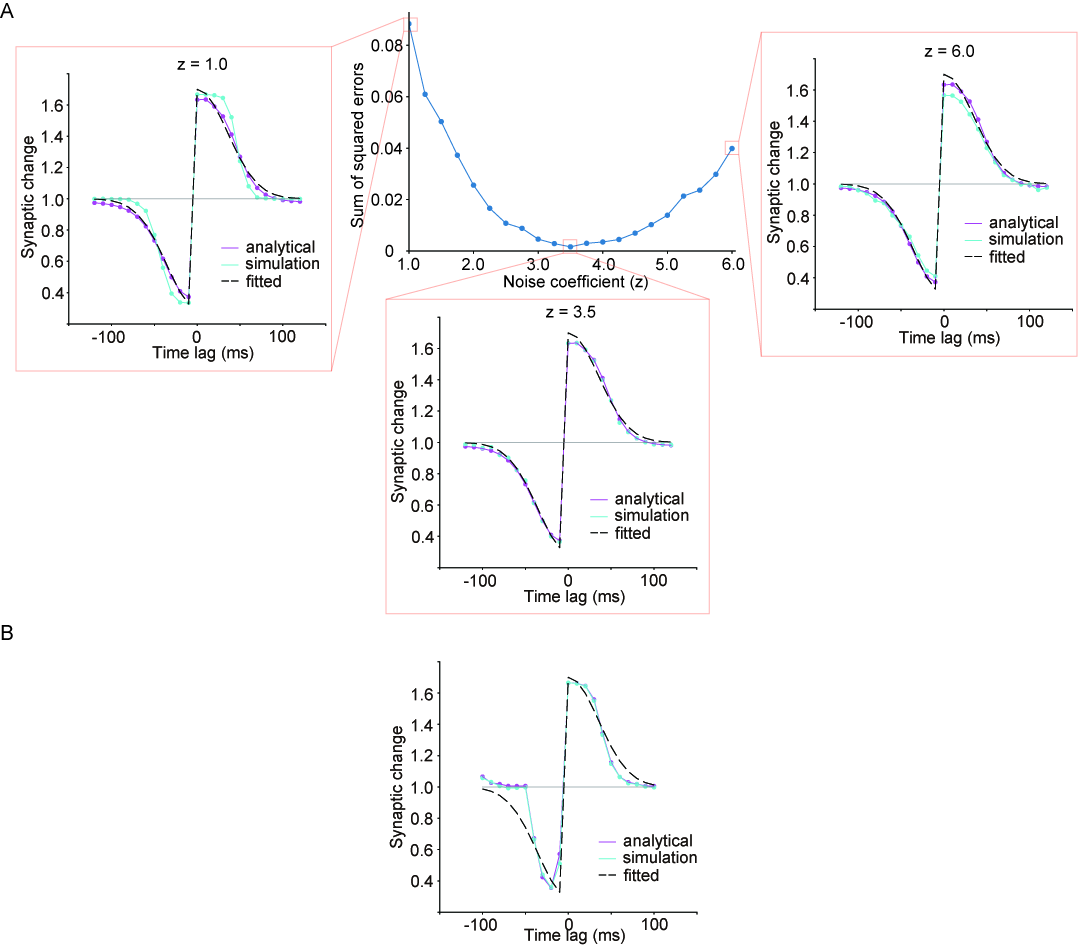

Supplement: S17 Fig — (A) The sum of squared errors (SSE) between analytical solutions and simulation results were calculated in different noise coefficient (a center figure) in simple model. All simulations were conducted in step size = 0.1. The simulation results were compared with analytical solutions and fitting curves (surrounding figures). The parameter set for STDP learning rule is shown in S8 Table. (B) Comparison of analytical and simulation results in parameter search for STDP learning rule in a representative Hodgkin–Huxley-based network model bifurcated by the post-synaptic mechanism. The parameter set for channel or receptor conductance of network model was the same as in Fig 3C and the parameter set for STDP learning rule is shown in S8 Table. The simulation was conducted by step size = 0.1. (TIF) [file pbio.3003198.s017.tif]

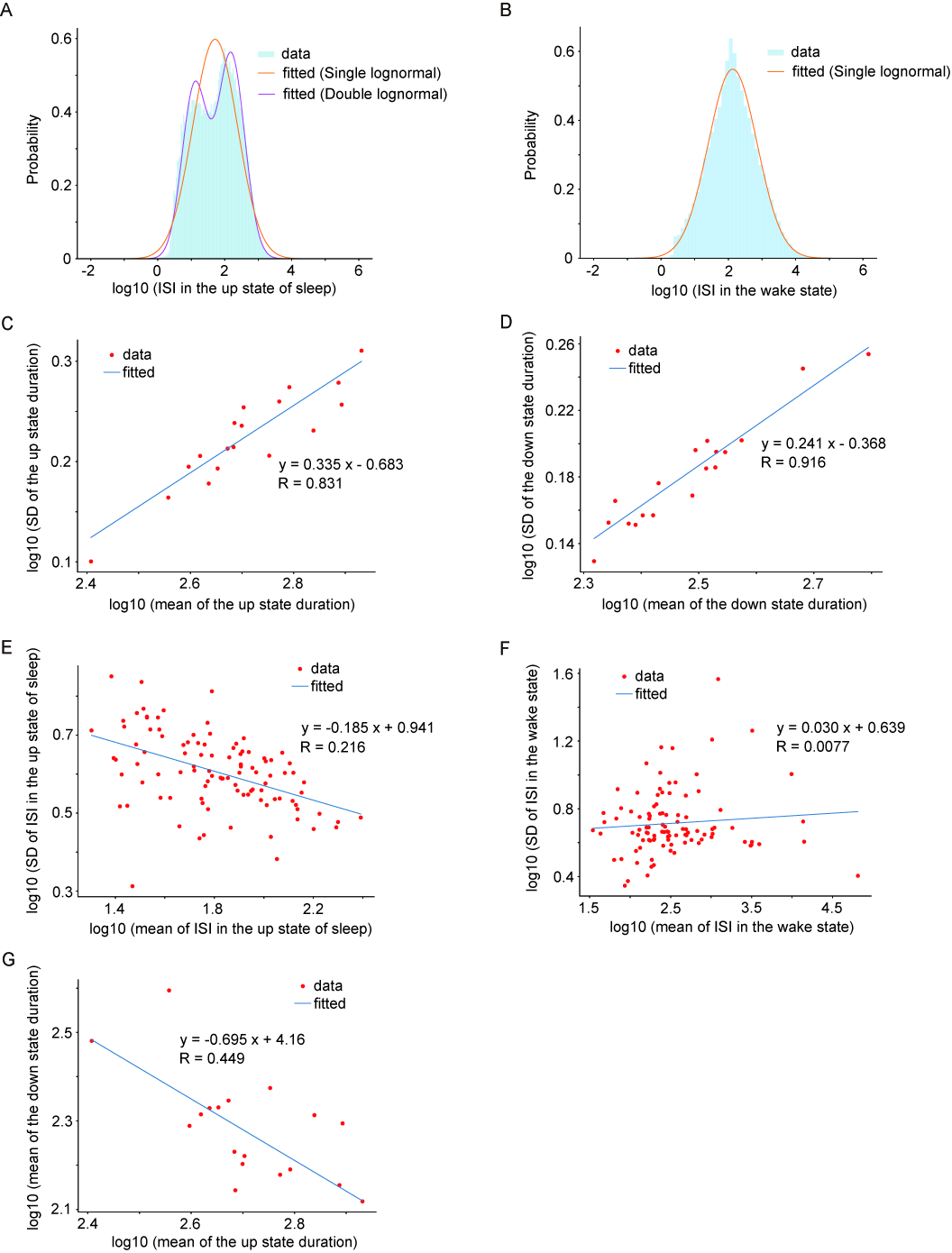

Supplement: S18 Fig — (A, B) The distribution for ISI of the spike-train data for all excitatory neurons (verified by cross-correlogram) in a dataset of a previous article [8,22]. Lognormal distributions for ISI in Up states in the state of sleep (A) and wake (B) are shown. The lognormal distributions fitted well in the ISI of the state of wake (B). Although mixed lognormal distributions were expected in the ISI of the sleep Up states, we assumed a single lognormal distribution in simulations for simplification (A). (C–G) We performed the linear regression analysis on the mean and SD of Up-state duration (C), the mean and SD of Down-state duration (D), the mean and SD of ISI in the state of wake (E) and in the Up states of sleep (F) and the mean Up-state duration and mean Down-state duration (G). (TIF) [file pbio.3003198.s018.tif]

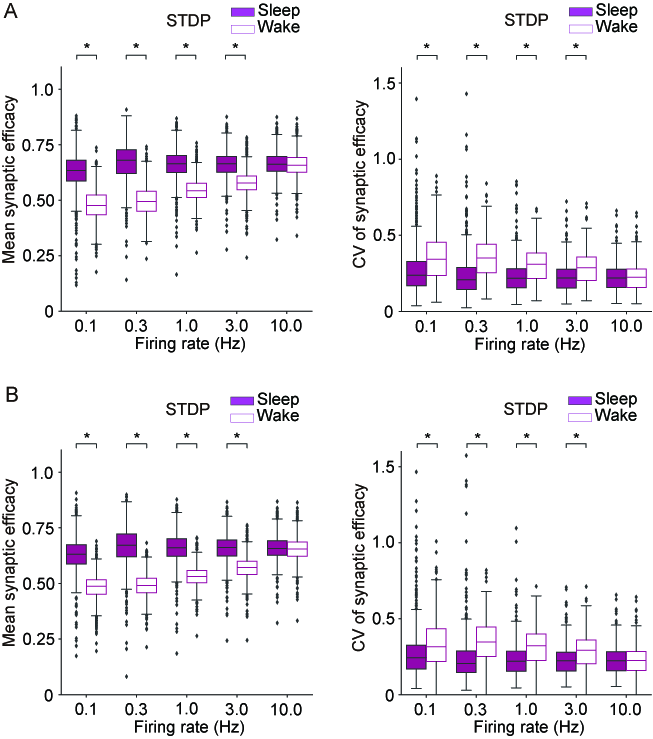

Supplement: S19 Fig — (A, B) Box plots for mean synaptic efficacy during sleep-like and wake-like firing patterns with 10 mV (A) and 5 mV (B) membrane potential differences are shown (n = 1,000 for each firing rate, n represents the number of synaptic learning rules). The parameter sets for STDP and spike trains are the same as in Fig 1H. The parameters for constructing waveforms are shown in S3 Table. Initial synaptic efficacies in all the synapses were 0.5 and synaptic efficacies were simulated for 6 min. Synaptic efficacies for the last 2 min were averaged and compared between sleep-like and wake-like firing patterns. The whiskers above and below of box plots show minimal to maximal values. The box extends from the 25th to the 75th percentile and the middle line indicates the median. Bayesian statistical analysis was performed using Markov Chain Monte Carlo method to infer posterior distributions of average differences in mean synaptic efficacy between sleep-like firing patterns and wake-like firing patterns. Asterisks (*) indicate 95% CIs do not include zero. The data underlying the graphs shown in the figure can be found in Tables T and U in S2 Data. The 95% CIs for the distributions of average differences are shown in Table K in S3 Data. (TIF) [file pbio.3003198.s019.tif]

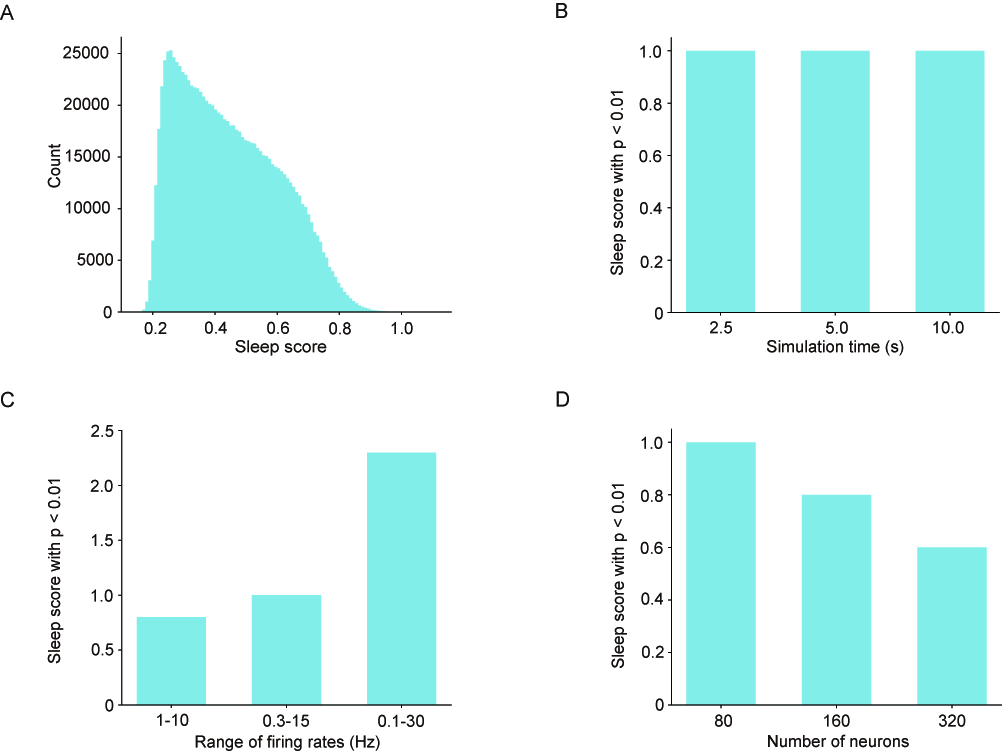

Supplement: S20 Fig — (A) Distribution of sleep scores in 1,000,000 desynchronized spikes in the condition of 80 neurons, 5 s simulation time and 0.5–15 Hz. Wake-like desynchronized spikes were sampled from lognormal distributions for ISI (see “Materials and methods, Definition of lognormal distributions based on in vivo recordings”). (B–D) sleep scores with p < 0.01 in different simulation times (B), ranges of mean firing rates (C) and number of neurons (D). P values were calculated by the distribution of 1,000,000 desynchronized spikes of each condition. The data underlying the graphs shown in the figure can be found in Table V in S2 Data. (TIF) [file pbio.3003198.s020.tif]
