# Supplementary material for: A unified framework to model synaptic dynamics during the sleep–wake cycle
Source: PLoS Biol. 2025 Jun 12;23(6):e3003198. doi: 10.1371/journal.pbio.3003198 (PMC12161594; doi:10.1371/journal.pbio.3003198)
Supplement: Attachment — Submitted filename: 150823_1_rebuttal_3240722_sjjjv2.docx [file pbio.3003198.s032.docx]

**Manuscript number:** RC-2024-02697

**Manuscript title:** Boundary conditions for synaptic homeodynamics during the sleep-wake cycle

**Authors:** Fukuaki L. Kinoshita, Rikuhiro G. Yamada, Koji L. Ode, Hiroki R. Ueda

**Corresponding author:** Hiroki R. Ueda

## 1. General Statements

**We thank all the reviewers for their time and their constructive criticism. We are encouraged by the overall positive and enthusiastic responses from the reviewers. We have taken all comments and suggestions seriously and revised the manuscript. These revisions include adding more explanation for the meaning of synaptic learning rules, language definitions, and model characteristics and limitations with more detailed figure legends. We are confident that we have addressed all the reviewer’s concerns by incorporating the reviewer’s suggestions into the revised manuscript. All changes are indicated in red font in the revised manuscript. The point-by-point response to all concerns raised by the reviewers follows. The line numbers indicated here refer to those in the revised manuscript.**

## 2. Point-by-point description of the revisions

**Reviewer #1**

**Evidence, reproducibility and clarity**

Summary:

While the function of sleep is still an unresolved mystery, some of the most influential theories propose that sleep serves a crucial role in regulating neuronal plasticity and synaptic strength. However, the exact way how synaptic strength is affected by sleep and impaired by sleep deprivation is a topic of much controversy and ongoing debate in the field of sleep research (SHY vs WISE). Using computation models, the manuscript illustrates that opposite effects of sleep on synaptic efficacy can be found, depending on the firing patterns and learning rules. Specifically, sleep promotes synaptic strength and efficacy under Hebbian and spike-timing dependent plasticity rules and it resulted in synaptic depression under anti-Hebbian and anti-STDP rules.


Major comments:

Introduction, line 64 and further: An important omission in the introduction is that several studies have shown that sleep deprivation, i.e., extended wakefulness, results in a loss of spines in some brain regions such as the hippocampus, which is directly opposing the SHY hypothesis (for review, see Raven et al. Sleep Med Rev 39: 3-11, 2018).

We appreciate the reviewer’s valuable comment. Indeed, as correctly pointed out, several studies have reported synaptic weakening in the hippocampus and cortical regions following sleep deprivation, which appears to contradict the SHY.

We have incorporated this point into the *introduction* section (lines 64-67), adding several articles, including Raven et al., the reviewer suggested.

Introduction, line 85-87: A short explanation of what exactly the anti-Hebbian and anti-STDP rules are, is important here. It may seem obvious to the authors, but it is best to spell it out for the potential broad readership interested in this paper.

We appreciate the reviewer’s important suggestion.

Previous studies reported that Anti-Hebbian plasticity, which leads to depression when synapses are presented with correlated activity, serves critical functions in the discrimination of specific spike sequences in the cortico-striatal synapses (G. Vignoud et al., Commun. Biol, 2024) and the detection of novel stimuli in mormyrid fish (P. D. Roberts et al., Biol. Cybern, 2008; P. D. Roberts et al, Front. Comput. Neurosci, 2010).

We have added the explanations for Anti-Hebbian and Anti-STDP rules into the *introduction* section (lines 87-89)

Results, line 116, 129/130, 333, 395, 400, figure captions: Pleases explain what is meant with the terms 'pre-neuronal synapse' and 'post-neuronal synapses'.

We appreciate the reviewer’s advice. We have replaced ‘pre-neuronal synapse’ and ‘post-neuronal synapse’ with ‘pre-synaptic’、’post-synaptic’, respectively, for readability in the *Results* section (lines 118-119, 131-133, 368, 371, 432, 436 and 437) and Figure legends.

Results, line 121-124 say that synaptic efficacy became higher in sleep-like states than in wake-like states under Hebbian and STDP learning rules and opposite results were observed with anti-Hebbian and anti-STDP learning rules. While these relative differences are indeed visible in Figure 1H, the figure also suggests that synaptic efficacy during sleep was largely independent of the average firing frequency. In other words, synaptic efficacy seems to be dependent on firing frequency only during wakefulness. Is that correct?

The reviewer raised an important point. As shown in Fig. 1H, synaptic efficacy during sleep appears to be largely independent of mean firing rates. Here, the firing rates were adjusted by varying Down-state durations. Regarding the relationship between firing patterns and synaptic efficacy, synaptic efficacy is influenced not only by firing frequency but also by how firing patterns are generated. When firing rates are adjusted by changing ISI, synaptic efficacy during sleep also increases with higher firing rates as wake-like patterns (Fig. 5). In Fig. 2D and E, we demonstrated that the synaptic efficacy during sleep becomes higher than during wakefulness regardless of whether the spike patterns were generated with changing Down-state duration or ISI, assuming the same mean firing rates during the sleep-like and wake-like states. We have clarified this point by adding the explanation in the *Discussion* section (lines 318-323)

Results, line 199 and down model the effect of differences in mean firing rate between sleep and waking, which is a crucial addition and more realistic approach for most brain regions that have lower average firing rates during sleep. It is interesting that in this case the relative effects of sleep and wakefulness can change direction, depending on the average firing frequency. Would the authors argue that this may even result in opposite effects in different brain regions after waking or sleep deprivation?

We appreciate the reviewer raising the interesting point. Our model predicted that the direction of synaptic changes depends on learning rules and firing rates. This prediction indicated that different brain regions may exhibit synaptic changes even in opposite directions after prolonged wakefulness or sleep deprivation. For example, under Hebbian and STDP, our model predicted that brain regions with firing rates increased during wakefulness or sleep deprivation compared to sleep would follow SHY, while brain regions where firing rates remain unchanged or decreased compared to sleep would follow WISE. The experimental validation of these predictions, focusing on brain regions with different activation states during wakefulness, is an interesting future work. We have clarified this point into the *Discussion* section (lines 260-262).

Figure 1: The caption needs more details to help understand the different panels. some work. (B) What is a post-neuronal synapse? (C) How exactly is synaptic efficacy defined? (E) Not totally clear what the colored top panels represent.

We sincerely appreciate the reviewer’s thoughtful feedback. We agreed that Figure 1 required a more thorough explanation. In response, we have expanded the figure legend to provide more detailed information for readers to easily understand.

Figure 5B. Since this appears to be a graphical abstract and unified framework for all the modelled parameters and learning rules, should this not be a separate figure?

We thank the reviewer for the helpful suggestion. We have renumbered Figure 5B as Figure 6.

Figures captions: The information provided in the figure captions is in many cases quite minimal and does not reflect the complexity of some of the figure panels. This often makes it hard for a reader to extract all the relevant information without thumbing back and forth between figures, captions and main text. I strongly suggest to add more detail to the figure captions to make them more stand-alone and self-explanatory.

We sincerely appreciate the reviewer’s significant feedback. We have added detailed explanations in the figure legends, including Supplementary Figures, for readers to understand easily.

Reviewer #1 (Significance (Required)):

This paper addresses a major controversy in the field of sleep research: does sleep strengthen neuronal connections in the brain or does it downscale and weaken them (Raven et al. 2018)? Using computation models, the current paper shows that both options are possible and it does an admirable job in bridging the different views on sleep and synaptic strength. As such, the conceptual value of this paper can hardly be overestimated and provides an important framework for future experimental studies.

This paper is of interest for most everybody interested in sleep and brain function, as well as neuroscientist with a broader interest in brain plasticity.

**Reviewer #2**

**Evidence, reproducibility and clarity**

I am not qualified to review this manuscript because I'm not sufficiently familiar with the type of modelling performed here and the specific use of terms. For example, without providing any explanation, I cannot reconstruct whether the estimates of synaptic efficacy (eq.1) are valid and applicable to the questions asked.
I do have 2 general comments. I do find the premise of WISE intriguing and understand the attractiveness of the idea of opposing 'WISE' to SHY. Nevertheless, SHY is a theory that does not discount the occurrence of synaptic strengthening during sleep. It is rather that during sleep there is a net down-scaling. Therefore, the assumptions, as they are presented here, are confusing the issue.

We are deeply grateful that the reviewer found WISE intriguing and appreciate the insightful comment. We agree that SHY does not deny the occurrence of synaptic strengthening during sleep, but rather proposes a net downward scaling under the assumption of the overall synaptic homeostasis. In the present study, we assumed that **SHY** describes a **net downscaling** during sleep (and does not deny the occurrence of synaptic strengthening of some synapses during sleep) while **WISE** describes **a net upscaling during sleep** (and does not deny the occurrence of synaptic weakening of some synapses during sleep). **Both SHY and WISE fulfill synaptic homeostasis.** For example, **SHY upscales** synaptic strength **during wakefulness** and **downscales during sleep** to achieve synaptic homeostasis. On the other hand, **WISE upscales** synaptic strength **during sleep** and **downscales during wakefulness** to achieve synaptic homeostasis. Our study demonstrated that **WISE is compatible with Hebbian and STDP** learning rules when average neuron firing frequency is similar between sleep and wakefulness, and SHY is not compatible with Hebbian and STDP learning rules, but rather **compatible with** **Anti-Hebbian** and **Anti-STDP** learning rules.

We agreed with the reviewer that the lack of an explicit definition of SHY and WISE in the context of the present study could cause confusion for readers. Therefore, we have added a sentence to clarify SHY and WISE in the present study in the first paragraph of the *Results* section (lines 127-128), specifically defining them in terms of relative net synaptic changes within local neural network.

SHY was, in part, inspired by a type of plasticity that is not considered here, namely synaptic homeostasis. Would adding such a mechanism to the model alter any of the predictions?"

We appreciate the reviewer raising an important point on synaptic homeostasis. In this study, we did not explicitly include synaptic homeostasis in the preposition but **consider synaptic homeostasis in the definitions of SHY and WISE**. For example, we assume that **SHY upscales** synaptic strength **during wakefulness** and **downscales during sleep** to achieve synaptic homeostasis while **WISE upscales** synaptic strength **during sleep** and **downscales during wakefulness** to achieve synaptic homeostasis. Importantly, since **both SHY and WISE can achieve synaptic homeostasis**, there are two types of synaptic homeostasis. In our study, **WISE-type synaptic homeostasis** is compatible with **Hebbian and STDP** learning rules when average neuron firing frequency is similar between sleep and wakefulness, and **SHY-type synaptic homeostasis** is **compatible with** **Anti-Hebbian** and **Anti-STDP** learning rules. Since our studies already consider two types of synaptic homeostasis, adding the further mechanism of synaptic homeostasis in the preposition would not alter our predictions. We described these points in the *Model characteristics and limitations* part in the *Discussion* section (lines 332-339).

Reviewer #2 (Significance (Required)):

I do find the premise of WISE intriguing and understand the attractiveness of the idea of opposing 'WISE' to SHY. Nevertheless, SHY is a theory that does not discount the occurrence of synaptic strengthening during sleep. It is rather that during sleep there is a net down-scaling. Therefore, the assumptions, as they are presented here, are confusing the issue. SHY was, in part, inspired by a type of plasticity that is not considered here, namely synaptic homeostasis. Would adding such a mechanism to the model alter any of the predictions?"

**Reviewer #3 (Evidence, reproducibility and clarity (Required)):**

Understanding the functions of sleep has and remains a key question in neuroscience. A popular hypothesis is that sleep is fundamental to learning and memory and that this can be detected and measured at the level of neural networks and connections as increased synaptic weights across waking states and reduced synaptic weights or depression during sleep states. However, there are many contradictions in the literature and while it is accepted that sleep plays a role in memory consolidation, the molecular/cellular basis of this is far from clear. As considerable experimental data on synaptic function have been collected during sleep and wake states, here the authors turned to modelling how manipulating the rules of synaptic plasticity can illuminate the problem. In this manuscript, the authors report the outcomes of these simulations neuronal oscillations, firing, and synaptic plasticity across sleep-like and wake-like neural states. They report that their simulations can account for several irregularities and highlight differential involvement of spike-firing dependent plasticity (STDP) and anti-STDP in wake and NREM sleep. In particular they note that under Hebbian and STDP rules, firing patterns associated with wake lead to decreased synaptic weights, while sleep-like patterns bolster synaptic weights and collectively they describe this tendency as WISE. They also note that under Anti-Hebbian and Anti-STDP rules, synaptic depression was observed under NREM. The chief strength of this study is shows how simulation can aid in bringing together disparate observations into a well-worked study space.

This is a well-written manuscript that is easily to follow and amply illustrated. The study seems very exciting but unfortunately I am not a mathematician so I cannot attest to the veracity or originality of the model. Assuming it is robust, it does appear to account for a quite a few anomalies (and inaccuracies depicted in textbooks). It would be helpful to discuss the limitations of other models that have been suggested to synaptic functions of sleep.

We appreciated the reviewer’s constructive suggestions. Some computational studies have investigated synaptic changes in neural networks under STDP protocols using Ca^2+^-based plasticity models (M. Graupner et al., PNAS, 2012; G. Chindemi et al., Nat. Commun, 2022), while other studies have examined how SWO affects synaptic plasticity under STDP conditions (T. Tadros et al., J.Neurosci, 2022). However, these previous studies were limited to a single synaptic learning rule or firing pattern. Our study is the first to comprehensively investigate synaptic dynamics during the sleep-wake cycle by integrating a Ca^2+^-based plasticity model to represent various types of synaptic learning rules and various simulated sleep-wake firing patterns.

We have added the sentences related to the reviewer’s comments in the *Model characteristics and limitations* part in the *Discussion* section (lines 306-312).

Much of the neurophysiological data comes from recordings in rodents, so the model is simulating rat EEG signatures-how readily applicable is this to the human condition? Indeed, how readily can they compare between mouse and rat? The authors should expand on this in the discussion section.

Another potential weakness or limitation is the unanswered question of the model can account for sleep/wake changes in other areas of the cortex or thalamus etc.

Does this model apply equally to males and females?

We appreciate the reviewer for raising this significant point. As the reviewer pointed out, we generated firing patterns using parameters derived from rat firing patterns (B. O. Watson et al., Neuron, 2016), such as ISI, Up-state duration, and Down-state duration. While we started our simulations from those parameter sets, we tested a range of different values for each parameter and found consistent results (detailed in *Supplementary Materials, Generation of sleep and wake-like firing patterns*). The ranges of Up-state and Down-state durations during SWO in mice, rats, and cats are approximately 100-500 milliseconds (M. Steriade et al., J. Neurophysiol, 2001; V. Crunelli et al., Pflugers Arch, 2012), while in humans, Up-state durations range from 250-1000 milliseconds (B. A. Riedner et al., Sleep, 2007), all of which fall within the ranges examined in Figs. 2 *D* and *E*. Similarly, wake-state ISI across various species typically range from 2-100 milliseconds (M. Steriade et al., J. Neurophysiol, 2001; G. Maimon et al., Neuron, 2009), mostly within the scope covered in Fig. 2E. Therefore, we suppose our finding in the present study captured universal aspects of synaptic dynamic in the sleep and wake cycles regardless of species, brain region, or sex.

We have added the description in the *Model characteristics and limitations* part in the *Discussion* section (lines 312-331).

Minor typo: ref. 24 is missing page and volume numbers.

Thank you for pointing out this typo. We corrected this by adding the page and volume numbers in Ref. 28 in the revised manuscript.

Reviewer #3 (Significance (Required)):

As noted above, there are discrepancies in the literature regarding synaptic plasticity and its mechanisms across the sleep-wake cycle. This model appears to answer some of the reasons for these and provides a framework for further experimental research to interrogate these mechanisms.
