# Supplementary material for: A unified framework to model synaptic dynamics during the sleep–wake cycle
Source: PLoS Biol. 2025 Jun 12;23(6):e3003198. doi: 10.1371/journal.pbio.3003198 (PMC12161594; doi:10.1371/journal.pbio.3003198)
Supplement: Attachment — Submitted filename: Responses_to_reviewers_250422.docx [file pbio.3003198.s034.docx]

**Manuscript number:** PBIOLOGY-D-25-00188R1

**Manuscript title:** A unified framework to model synaptic dynamics during the sleep-wake cycle

**Authors:** Fukuaki L. Kinoshita, Rikuhiro G. Yamada, Koji L. Ode, Hiroki R. Ueda

**Corresponding author:** Hiroki R. Ueda

## 1. General Statements

**We thank all the editors and reviewers for their time, suggestions, and constructive criticism. We are encouraged by the overall positive and enthusiastic responses from the reviewers. We have taken all comments and suggestions seriously and revised the manuscript again. These revisions include adding more explanation for model characteristics and limitations. We are confident that we have addressed all the editors’ and reviewers’ concerns in the revised manuscript. All changes are indicated in red font in the revised manuscript. The point-by-point response to all concerns raised by the editors and reviewers follows. The line numbers indicated here refer to those in the revised manuscript.**

## 2. Point-by-point description of the revisions

Editorial requests:

* We would like to suggest a different title to improve its accessibility for our broad audience: "A unified framework to model synaptic dynamics during the sleep-wake cycle"

We really appreciate the editorial significant suggestions. We have changed the title from “Boundary conditions for synaptic homeodynamics during the sleep-wake cycle” to "A unified framework to model synaptic dynamics during the sleep-wake cycle."

* Please add the links to the funding agencies in the Financial Disclosure statement in the manuscript details.

We added links to the funding agencies in the *Funding* section of the manuscript.

* DATA POLICY:
You may be aware of the PLOS Data Policy, which requires that all data be made available without restriction: <http://journals.plos.org/plosbiology/s/data-availability>. For more information, please also see this editorial: <http://dx.doi.org/10.1371/journal.pbio.1001797>

Note that we do not require all raw data. Rather, we ask that all individual quantitative observations that underlie the data summarized in the figures and results of your paper be made available in one of the following forms:

1) Supplementary files (e.g., excel). Please ensure that all data files are uploaded as 'Supporting Information' and are invariably referred to (in the manuscript, figure legends, and the Description field when uploading your files) using the following format verbatim: S1 Data, S2 Data, etc. Multiple panels of a single or even several figures can be included as multiple sheets in one excel file that is saved using exactly the following convention: S1_Data.xlsx (using an underscore).

2) Deposition in a publicly available repository. Please also provide the accession code or a reviewer link so that we may view your data before publication.

Regardless of the method selected, please ensure that you provide the individual numerical values that underlie the summary data displayed in the following figure panels as they are essential for readers to assess your analysis and to reproduce it: 1H, 2ABC, 3EF, 4GH, 5, S2, S3, S4, S8, S9, S10, S11, S12FG, S13FG, S14, S15, S16, S19 and S20.
NOTE: the numerical data provided should include all replicates AND the way in which the plotted mean and errors were derived (it should not present only the mean/average values).
Please also ensure that figure legends in your manuscript include information on where the underlying data can be found, and ensure your supplemental data file/s has a legend.
Please ensure that your Data Statement in the submission system accurately describes where your data can be found.

We made S1_Data.xlsx file to provide the individual numerical values that underlie the summary data displayed in the following figure panels: 1H, 2ABC, 3EF, 4GH, 5, S2, S3, S4, S8, S9, S10, S11, S12FG, S13FG, S14, S15, S16, S19 and S20. We have ensured that these data are referred to in the main manuscript and figure legends, and the Description field.

* CODE POLICY

Per journal policy, if you have generated any custom code during the course of this investigation, please make it available without restrictions. Please ensure that the code is sufficiently well documented and reusable, and that your Data Statement in the Editorial Manager submission system accurately describes where your code can be found.

Please note that we cannot accept sole deposition of code in GitHub, as this could be changed after publication. However, you can archive this version of your publicly available GitHub code to Zenodo. Once you do this, it will generate a DOI number, which you will need to provide in the Data Accessibility Statement (you are welcome to also provide the GitHub access information). See the process for doing this here: <https://docs.github.com/en/repositories/archiving-a-github-repository/referencing-and-citing-content>

We archived our publicly available GitHub code to Zenodo <https://doi.org/10.5281/zenodo.15210933>.

We also described the data availability in the *Data Availability* in the manuscript (line 18-23).

* Please note that per journal policy, the model system/species studied should be clearly stated in the abstract of your manuscript.

In the present study, we selected parameter ranges according to previous articles that focused on mammalian cortical neurons. We clearly stated the model system/species we mainly investigated in the *Abstract* (line 60 and 65).

* Please move the supplementary methods and references to the main manuscript file.

We moved the supplementary methods and references to the main manuscript file.

Reviewer #1: In this manuscript, Kinoshita and colleagues present a new modelling approach and framework in which to understand the relationships among sleep/wake states, neuronal activity, rules for learning, and synaptic plasticity. Through their modelling of Hebbian and spike-timing dependent plasticity, they identified a tendency, wake inhibition and sleep excitation (WISE) in which firing patterns associated with wake decreased synaptic weights, while patterns of neuronal firing that resembled sleep strengthened synaptic weights. This differed from anti-Hebbian and anti-spike timing dependent plasticity in which synaptic depression was modelled under NREM sleep. This latter observation is akin to the synaptic homeostasis hypothesis. They further showed that these synaptic changes depended on firing rate differences between NREM and wakefulness.  Thus, there is considerable potential with this model/approach that could lead to a big change in how synaptic plasticity, neuronal activity, and learning are thought off.

The manuscript is much improved and the authors have attempted to address many of points raised previously.  The manuscript is improved by the authors altering their manuscript. There are still some questions that I think merit consideration.

1)      It is not entirely clear why the authors have assumed that CamKII is responsible for the homeostatic oscillation.  This needs to be better fleshed out. Are there other kinases or signaling pathways that are of similar importance?  How do the authors incorporate the different phosphorylation states of these enzymes? Similarly, it is not too obvious as to why the second state interacts through molecules that induce slow wave sleep oscillation.

The reviewer raised an important point. The reason why we focus on CaMKII is that it has multiple phosphorylation states, which are linked to sleep induction and maintenance mechanisms (Tone et al. 2022). If multiple phosphorylation states exist, oscillatory patterns with defined periodicity can emerge (Jolley, Ode, and Ueda 2012).  Moreover, previous studies demonstrated that the phosphorylated CaMKII increases proportionally to the extent of wakefulness (Brüning et al. 2019) and can directly or indirectly interact with downstream molecules that generate SWO (Tatsuki et al. 2016; Ode and Ueda 2020; Wang et al. 2022).

It is noteworthy that the homeostatic oscillation of CaMKII may be just one of many examples.  For example, salt-inducible kinase 3 (SIK3) and extracellular signal-regulated kinase (ERK) are alternative candidates because these kinases also have multiple phosphorylation sites and are related to circadian rhythm (Obrietan, Impey, and Storm 1998; Akashi and Nishida 2000; Vanderheyden et al. 2013; Funato et al. 2016; Hayasaka et al. 2017; Kim et al. 2022).

It is unclear which phosphorylation state of CaMKII highly correlates with Process S, or interacts with the molecules that induce SWO. Our model incorporated two principal phosphorylation states of CaMKII—designated as "first state" and "second state"—both potentially interacting with molecules to generate SWO and exhibiting positive correlations with Process S. In Fig S11A, we systematically conducted simulations with varying parameters across potential combinations. We selected one where a specific phosphorylation state exhibited maximal Pearson’s correlation coefficients with Process S. The results suggest that the first state (expecting CaMKII pT286/T287) demonstrates a stronger correlation with Process S, while the second state appears more intimately involved in generation of the SWO. These computational predictions require experimental validation in subsequent studies. We have added these statements in the *Model characteristics and limitations* part in the *Discussion* section of the manuscript (line 379-392).

2)      I am somewhat confused by the role of firing rate in these processes. In their rebuttal to reviewer 1, they emphasized that the mechanism via which firing patterns are generated is important. It could be argued that this also alters the types of neurochemicals being released as burst firing is often associated with peptide or hormone release which does not seem to be integrated into the model or types of synaptic plasticity. This should be expanded upon in the discussion.

We appreciate the reviewer’s valuable comments. As the reviewer noted, burst firing promotes the release of neuropeptides such as Substance P and Neuropeptide Y, which significantly contribute to the modulation of neural circuits (Whim and Lloyd 1989; Fernandez-Chiappe et al. 2021) . Although systematic investigations of the relationship between firing patterns and neurotransmitter release in cortical neurons remain limited, several studies have documented the release of neuropeptides, including Neuropeptide Y, somatostatin (SST), and vasoactive intestinal peptide (VIP), alter the thresholds in STDP in the cortex (Smith et al. 2020). In contrast with those of classical neurotransmitters, these neuropeptides predominantly exert their effects through G-protein coupled receptors, functioning over extended temporal domains, potentially introducing additional complexity to plasticity mechanisms (Casello et al. 2022). Our current computational framework does not explicitly incorporate these neuromodulatory dynamics for simplification. Elucidating the contributions of neuropeptides and hormones influenced by sleep-wake firing patterns to synaptic plasticity and homeostatic control of neural excitability in cortical neurons represents an important direction for subsequent studies. We have incorporated these important points in the *Model characteristics and limitations* part in the *Discussion* section (line 393-404).

3)      The range of duration in up and down states across species is broad but it is difficult to see how the authors have validated their model to support these assumptions. Please expand.

Indeed, as the reviewer correctly noted, the duration of Up and Down states exhibits some variation across species. Based on our comprehensive literature review, the Up- and Down-state durations in most mammalian species are approximately 100-1000 milliseconds (Steriade, Timofeev, and Grenier 2001; Riedner et al. 2007; Crunelli et al. 2012), almost all of which fall within the range we examined in Fig 2D and 2E. These figures demonstrate that our principal finding—WISE under STDP when average firing rates during sleep and wakefulness are equivalent—remains robust across a substantially diverse range of sleep-like firing patterns. We described these considerations in the *Model characteristics and limitations* part in the *Discussion* section (line 348-358).

Minor point:

Page 4, line 86.  Replace 'Reverse types of them...' with 'Reverse types of these...' and replace These at the start of  the next sentence (line 87) with Such.

We appreciate the reviewer’s advice. We have replaced “Reverse types of them” with “Reverse types of these” and replaced “These” at the start of the next sentence with “Such” (line 118-121).

Reviewer #2: Kinoshita et al

I was not one of the original reviewers of the paper, so I feel I should not be too critical to avoid 'double jeopardy' for the authors.

Overall, the study seems competently carried out. I think without further determination of in vivo plasticity rules under awake and sleep states, the conclusions remain a bit tentative, but they set a decent baseline for subsequent studies.

- the authors should be more explicit about the brain area they model.

We appreciate the reviewer’s suggestion, which aligns with the editorial recommendation. We have constrained our parameter space to encompass physiologically plausible ranges for cortical neurons by referring to previous literature (Graupner and Brunel 2012; Tatsuki et al. 2016). For the simple model used in Figs 1, 2, and 5A, the sleep-like and wake-like firing patterns were derived from the records in the rat prefrontal cortex (PFC) (Watson et al. 2016).

We have clarified the brain area we focused on in this study in the *Abstract* section of the manuscript (line 60 and 65).

- Fig1, what is the mean efficacy before stimulation?

The reviewer raised an important point. Mean efficacy before stimulation represents mean synaptic efficacy in the initial states. Because the initial synaptic efficacies of all synapses are 0.5 in the setting of the first simple model, the mean efficacy before stimulation is 0.5.

We added the explanatory sentence in the Fig 1 legend.

- I should also note that formally, the logic of drawing many models is slightly flawed.

Afterall, biology could have chosen a very particular setting, so that the outcome is not at all well described by the mean of many random choices.

We appreciate the reviewer’s significant comments. We agree that it is important to investigate the synaptic plasticity and function of networks in particular species, brain regions, and environments. However, synaptic plasticity can vary across species, brain regions and environments, and this heterogeneity likely underlies the contradictions between previous studies regarding synaptic changes during the sleep-wake cycle. In this study, we aimed to obtain more robust conclusions that are not limited to particular species or environments. Therefore, we comprehensively investigated the boundary conditions governing synaptic changes during the sleep-wake cycle with a lot of random parameter sets about firing patterns and synaptic learning rules. Because our intentions might not have been clear, we have explicitly elaborated on the objectives of our study in the Introduction section (Iine 106-111). We appreciate the reviewer’s significant comments, which are greatly valuable for this paper.

- there are many cases where variables are not named. This should be corrected and every variable should be defined (for an example see Hodgkin Huxley 1952)

The section on NMDA is particularly confusing. x_nmda has a DE but it is not used above it which uses x. But what even is it? It has a remarkably short time-scale.

If it is really that short, it could be omitted and the value replaced with its steady state.

The section on homeostasis (l444) is also particularly poor.

We appreciate the reviewer’s important suggestions and questions. We have defined variables, especially in the Hodgkin-Huxley-based model and sleep-wake dynamics model in the *Materials and Methods* section (line 531-591, 727-733, and 750-751). We have revised a differential equation of (Eq. 5) the reviewer pointed out because it was wrong.

The NMDA receptor kinetics are characterized by two differential equations (Eq. 5 and Eq. 6) derived from the original mathematical formulation (Tatsuki et al. 2016). X_nmda in our model has a smaller time constant than that in the original model to represent a more diverse range of synaptic learning rules. We have added the explanations in the Materials and Methods section (line 416-418).

- l132. Why is this called a simplified model? In the network in Fig2B, which neurons received the external input?

We appreciate the reviewer’s valuable question. We removed the word “simply” because it can make readers misunderstand that the model with 96 neurons is a simplified version (line 163-165). In the setting in Fig 2B, the connection patterns of neurons are different from each other. The schematic illustration in Fig 2B shows only one example. For example, in the schematic illustration, neurons 2 receives input from neuron 4, and neuron 7 receives input from neuron 2 and 6. We have redesigned the edges between neurons in Fig 2B to clarify the input-output relationships.

- if the authors continue this work, they might be interested in:

A stochastic model of hippocampal synaptic plasticity with geometrical readout of enzyme dynamics YE Rodrigues, CM Tigaret, H Marie, C O'Donnell, R Veltz Elife 12, e80152

Thank you for introducing this interesting paper. We appreciate the reviewer’s suggestion and intend to incorporate these insights into our future modeling.

- I'm not a proponent of using t-test for modelling results (after all, once one runs sufficient simulations, everything, apart from trivial cases, will be significant...)

We really appreciate the reviewer’s constructive comments. As the reviewer has pointed out, an extensive number of simulations increased the probability of detecting statistical significance.

Therefore, we tested the difference in mean synaptic efficacy and synaptic CV between two groups that follow normal distributions using Bayesian statistics in figure panels with a large number of simulation results. The analysis was performed according to the following steps:

Non-informative priors were set for each group's mean and standard deviation and the likelihood for the observed data was defined. A parameter representing the difference in averages (diff = mu1 - mu2) (mu1 and mu2 is a mean in each group) was defined. Then, the Markov Chain Monte Carlo method was used to sample from the posterior distribution. The sampling results were examined using a 95% credible interval (CI) to evaluate whether there was a significant difference between the two groups (i.e., whether zero was contained within the interval). We added the description of methods for Bayesian statistical analysis in the *Materials and Methods* section (line 802-813). We marked with an asterisk (*) in the comparisons of synaptic efficacy between sleep-like and wake-like patterns where the 95% CI did not include zero in Figs 1H, 2A-C, 5, S2, S3, S16A, and S19. We also showed the 95% CIs in S2_Data.

**References**

Akashi, M., and E. Nishida. 2000. “Involvement of the MAP Kinase Cascade in Resetting of the Mammalian Circadian Clock.” *Genes & Development* 14 (6): 645–49.

Brüning, Franziska, Sara B. Noya, Tanja Bange, Stella Koutsouli, Jan D. Rudolph, Shiva K. Tyagarajan, Jürgen Cox, Matthias Mann, Steven A. Brown, and Maria S. Robles. 2019. “Sleep-Wake Cycles Drive Daily Dynamics of Synaptic Phosphorylation.” *Science* 366 (6462): eaav3617.

Casello, Sanne M., Rodolfo J. Flores, Hector E. Yarur, Huikun Wang, Monique Awanyai, Miguel A. Arenivar, Rosario B. Jaime-Lara, Hector Bravo-Rivera, and Hugo A. Tejeda. 2022. “Neuropeptide System Regulation of Prefrontal Cortex Circuitry: Implications for Neuropsychiatric Disorders.” *Frontiers in Neural Circuits* 16 (June): 796443.

Crunelli, Vincenzo, Magor L. Lörincz, Adam C. Errington, and Stuart W. Hughes. 2012. “Activity of Cortical and Thalamic Neurons during the Slow (<1 Hz) Rhythm in the Mouse in Vivo.” *Pflugers Archiv: European Journal of Physiology* 463 (1): 73–88.

Fernandez-Chiappe, Florencia, Lia Frenkel, Carina Celeste Colque, Ana Ricciuti, Bryan Hahm, Karina Cerredo, Nara Inés Muraro, and María Fernanda Ceriani. 2021. “High-Frequency Neuronal Bursting Is Essential for Circadian and Sleep Behaviors in Drosophila.” *The Journal of Neuroscience: The Official Journal of the Society for Neuroscience* 41 (4): 689–710.

Funato, Hiromasa, Chika Miyoshi, Tomoyuki Fujiyama, Takeshi Kanda, Makito Sato, Zhiqiang Wang, Jing Ma, et al. 2016. “Forward-Genetics Analysis of Sleep in Randomly Mutagenized Mice.” *Nature* 539 (7629): 378–83.

Graupner, Michael, and Nicolas Brunel. 2012. “Calcium-Based Plasticity Model Explains Sensitivity of Synaptic Changes to Spike Pattern, Rate, and Dendritic Location.” *Proceedings of the National Academy of Sciences of the United States of America* 109 (10): 3991–96.

Hayasaka, Naoto, Arisa Hirano, Yuka Miyoshi, Isao T. Tokuda, Hikari Yoshitane, Junichiro Matsuda, and Yoshitaka Fukada. 2017. “Salt-Inducible Kinase 3 Regulates the Mammalian Circadian Clock by Destabilizing PER2 Protein.” *ELife* 6 (December). https://doi.org/10.7554/eLife.24779.

Jolley, Craig C., Koji L. Ode, and Hiroki R. Ueda. 2012. “A Design Principle for a Posttranslational Biochemical Oscillator.” *Cell Reports* 2 (4): 938–50.

Kim, Staci J., Noriko Hotta-Hirashima, Fuyuki Asano, Tomohiro Kitazono, Kanako Iwasaki, Shinya Nakata, Haruna Komiya, et al. 2022. “Kinase Signalling in Excitatory Neurons Regulates Sleep Quantity and Depth.” *Nature* 612 (7940): 512–18.

Obrietan, K., S. Impey, and D. R. Storm. 1998. “Light and Circadian Rhythmicity Regulate MAP Kinase Activation in the Suprachiasmatic Nuclei.” *Nature Neuroscience* 1 (8): 693–700.

Ode, Koji L., and Hiroki R. Ueda. 2020. “Phosphorylation Hypothesis of Sleep.” *Frontiers in Psychology* 11 (October): 575328.

Riedner, Brady A., Vladyslav V. Vyazovskiy, Reto Huber, Marcello Massimini, Steve Esser, Michael Murphy, and Giulio Tononi. 2007. “Sleep Homeostasis and Cortical Synchronization: III. A High-Density EEG Study of Sleep Slow Waves in Humans.” *Sleep* 30 (12): 1643–57.

Smith, Stephen J., Michael Hawrylycz, Jean Rossier, and Uygar Sümbül. 2020. “New Light on Cortical Neuropeptides and Synaptic Network Plasticity.” *Current Opinion in Neurobiology* 63 (August): 176–88.

Steriade, M., I. Timofeev, and F. Grenier. 2001. “Natural Waking and Sleep States: A View from inside Neocortical Neurons.” *Journal of Neurophysiology* 85 (5): 1969–85.

Tatsuki, Fumiya, Genshiro A. Sunagawa, Shoi Shi, Etsuo A. Susaki, Hiroko Yukinaga, Dimitri Perrin, Kenta Sumiyama, et al. 2016. “Involvement of Ca(2+)-Dependent Hyperpolarization in Sleep Duration in Mammals.” *Neuron* 90 (1): 70–85.

Tone, Daisuke, Koji L. Ode, Qianhui Zhang, Hiroshi Fujishima, Rikuhiro G. Yamada, Yoshiki Nagashima, Katsuhiko Matsumoto, et al. 2022. “Distinct Phosphorylation States of Mammalian CaMKIIβ Control the Induction and Maintenance of Sleep.” *PLoS Biology* 20 (10): e3001813.

Vanderheyden, William M., Jason R. Gerstner, Anne Tanenhaus, Jerry C. Yin, and Paul J. Shaw. 2013. “ERK Phosphorylation Regulates Sleep and Plasticity in Drosophila.” *PloS One* 8 (11): e81554.

Wang, Yuyang, Yoichi Minami, Koji L. Ode, and Hiroki R. Ueda. 2022. “The Role of Calcium and CaMKII in Sleep.” *Frontiers in Systems Neuroscience* 16 (December): 1059421.

Watson, Brendon O., Daniel Levenstein, J. Palmer Greene, Jennifer N. Gelinas, and György Buzsáki. 2016. “Network Homeostasis and State Dynamics of Neocortical Sleep.” *Neuron* 90 (4): 839–52.

Whim, M. D., and P. E. Lloyd. 1989. “Frequency-Dependent Release of Peptide Cotransmitters from Identified Cholinergic Motor Neurons in Aplysia.” *Proceedings of the National Academy of Sciences of the United States of America* 86 (22): 9034–38.
